# CD47 prevents the elimination of diseased fibroblasts in scleroderma

**DOI:** 10.1172/jci.insight.140458

**Published:** 2020-08-20

**Authors:** Tristan Lerbs, Lu Cui, Megan E. King, Tim Chai, Claire Muscat, Lorinda Chung, Ryanne Brown, Kerri Rieger, Tyler Shibata, Gerlinde Wernig

**Affiliations:** 1 Department of Pathology,; 2 Institute for Stem Cell Biology and Regenerative Medicine, and; 3 Division of Immunology and Rheumatology, Department of Medicine, Stanford School of Medicine, Stanford, California, USA.

**Keywords:** Autoimmunity, Dermatology, Fibrosis, Immunotherapy

## Abstract

Scleroderma is a devastating fibrotic autoimmune disease. Current treatments are partly effective in preventing disease progression but do not remove fibrotic tissue. Here, we evaluated whether scleroderma fibroblasts take advantage of the “don’t-eat-me-signal” CD47 and whether blocking CD47 enables the body’s immune system to get rid of diseased fibroblasts. To test this approach, we used a *Jun*-inducible scleroderma model. We first demonstrated in patient samples that scleroderma upregulated transcription factor JUN and increased promoter accessibilities of both JUN and CD47. Next, we established our scleroderma model, demonstrating that *Jun* mediated skin fibrosis through the hedgehog-dependent expansion of CD26^+^Sca1^–^ fibroblasts in mice. In a niche-independent adaptive transfer model, JUN steered graft survival and conferred increased self-renewal to fibroblasts. In vivo, JUN enhanced the expression of CD47, and inhibiting CD47 eliminated an ectopic fibroblast graft and increased in vitro phagocytosis. In the syngeneic mouse, depleting macrophages ameliorated skin fibrosis. Therapeutically, combined CD47 and IL-6 blockade reversed skin fibrosis in mice and led to the rapid elimination of ectopically transplanted scleroderma cells. Altogether, our study demonstrates the efficiency of combining different immunotherapies in treating scleroderma and provides a rationale for combining CD47 and IL-6 inhibition in clinical trials.

## Abstract 2

Combined CD47 and IL6 inhibition eliminates diseased fibroblasts and reverses skin fibrosis in a murine scleroderma model.

## Graphical abstract

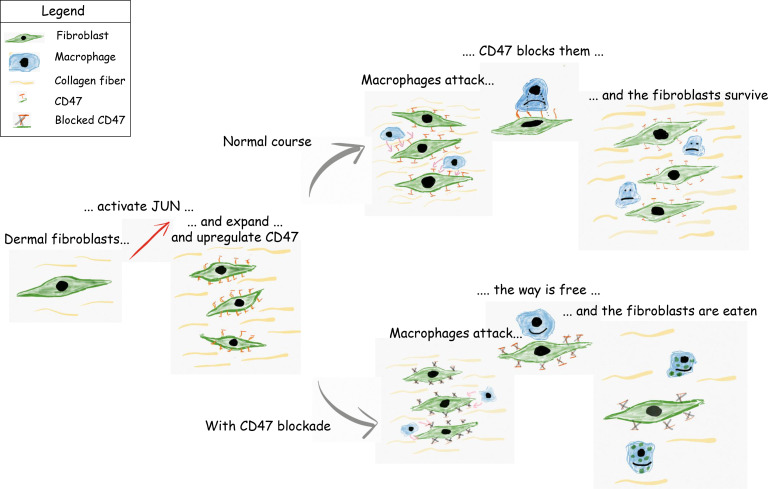

## Introduction

Scleroderma is the fibrotic skin manifestation of the autoimmune diseases morphea and systemic sclerosis, causing significant morbidity. Current treatments rely on immune suppression and primarily aim at stopping or slowing down the progression of scleroderma (1, 2). Unfortunately, these treatments only bring temporary relief to most patients. Therefore, we aimed at exploring a therapeutic approach that could not only prevent but also reverse the fibrotic skin changes. Considering that scleroderma leads to the accumulation of diseased fibroblasts, we hypothesized that the inhibition of the “don’t-eat-me-signal” CD47 would help the body’s immune system remove abnormal fibroblasts. This therapeutic approach not only would stop the disease as immune suppression does but also could permit its remission, thereby offering a breakthrough for the treatment of scleroderma.

Next to changes in the immune and vascular system, skin fibrosis represents one of scleroderma’s clinical symptoms (3–5). Fibrosis is marked by an excessive amount of connective tissue primarily formed by fibroblasts. One of the genes that fibroblasts commonly upregulate in several fibrotic conditions, especially idiopathic pulmonary fibrosis, is the transcription factor *JUN*, as our group has recently shown (6, 7). *JUN*, which belongs to the family of the AP-1 transcription factors and is activated through phosphorylation (phosphorylated JUN, p-JUN), otherwise contributes to malignant diseases and plays a role in different developmental programs (8, 9). To study the effects of *Jun*, our group uses a unique *Jun*-driven mouse model. In this model, *Jun* is inserted into the collagen locus and is under the control of a tetracycline-dependent promoter in the Rosa26 locus. Through doxycycline application, *Jun* can be induced either systemically through intraperitoneal injections or the drinking water or locally through injections. Another characteristic feature of fibroblasts is their phenotypic and functional diversity (10). During development, they form different lineages. Lineages can be distinguished by specific surface markers, and both CD26 and Sca1 are among these markers. While CD26^+^Sca1^–^ fibroblasts primarily reside in the upper dermis, CD26^–^Sca1^+^ fibroblasts can be mainly found in the lower dermis (10). Though not specifically explored in these subsets of fibroblasts, hedgehog signaling with its main effector, *Gli1*, has been shown to contribute to dermal fibrosis (11). Besides, hedgehog signaling plays a vital role in the development and the maintenance of stem cell populations (12, 13).

Immune therapy has changed the therapeutic landscapes for several cancers within a few years (14), unleashing the body’s own immune system in its fight against the tumor. Programmed cell death 1/programmed death ligand 1 (PD-1/PD-L1) inhibition boosts T cell function and is approved as a first-line therapy for various cancers, including non–small cell lung cancer (15, 16). CD47 prevents macrophages from eating their target cell, and CD47 inhibition is currently being investigated in advanced clinical trials (17, 18). In contrast to immune checkpoint inhibitors and “don’t-eat-me-signals,” interleukin-6 blockade aims at reducing inflammatory responses that can worsen autoimmune diseases (19).

Conceptually, this study aimed at investigating whether the “don’t-eat-me-signal” CD47 either alone or in combination with interleukin-6 blockade is able to prevent or reverse fibrotic skin changes in a *Jun*-driven mouse model. We hypothesized that this combination would allow the immune system to get rid of abnormal fibroblasts while preventing an ongoing inflammatory response through interleukin-6 (IL-6) blockade.

## Results

### Human scleroderma activates JUN and CD47.

**Figure 1:**
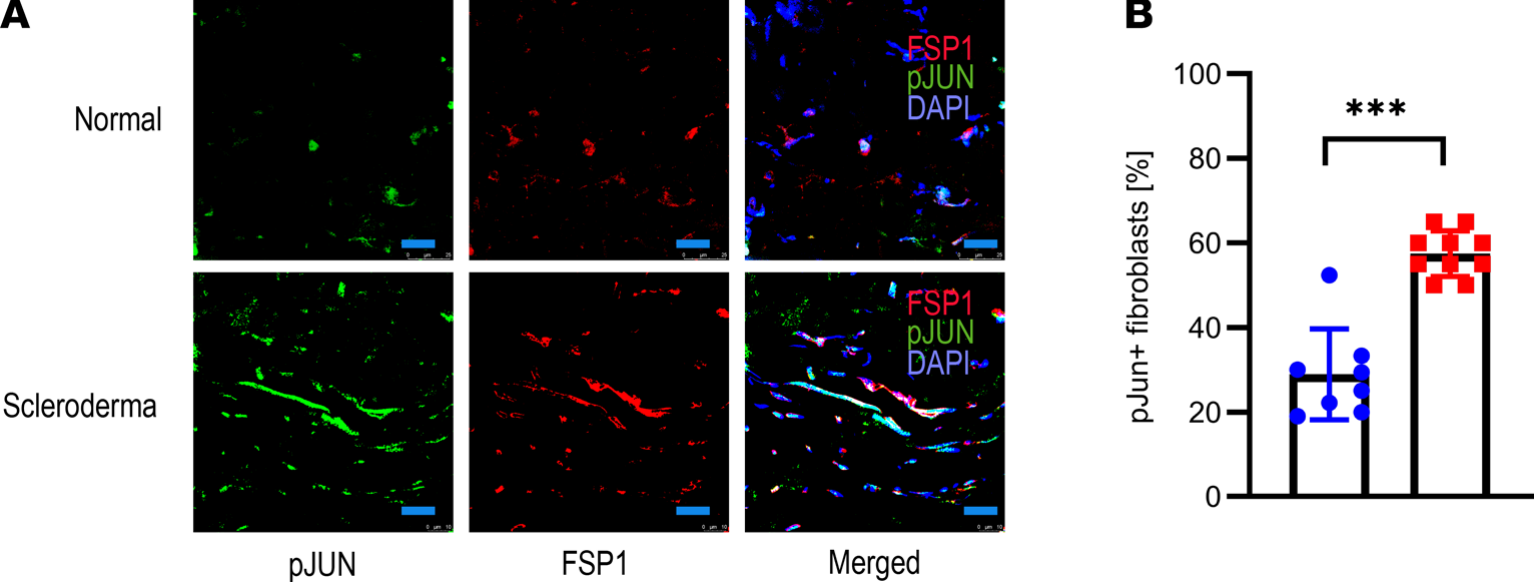
Human scleroderma upregulates JUN. (**A**) Immunofluorescence pictures of human scleroderma and normal skin stained against p-JUN and FSP1, counterstained with DAPI. Scale bar: 25 μm. *n* = 8–10. (**B**) Corresponding quantification of p-JUN^+^ fibroblasts. Two-sided *t* test. ***P < 0.001. *n* = 8–10. Bars represent means with standard deviations.

**Figure 2:**
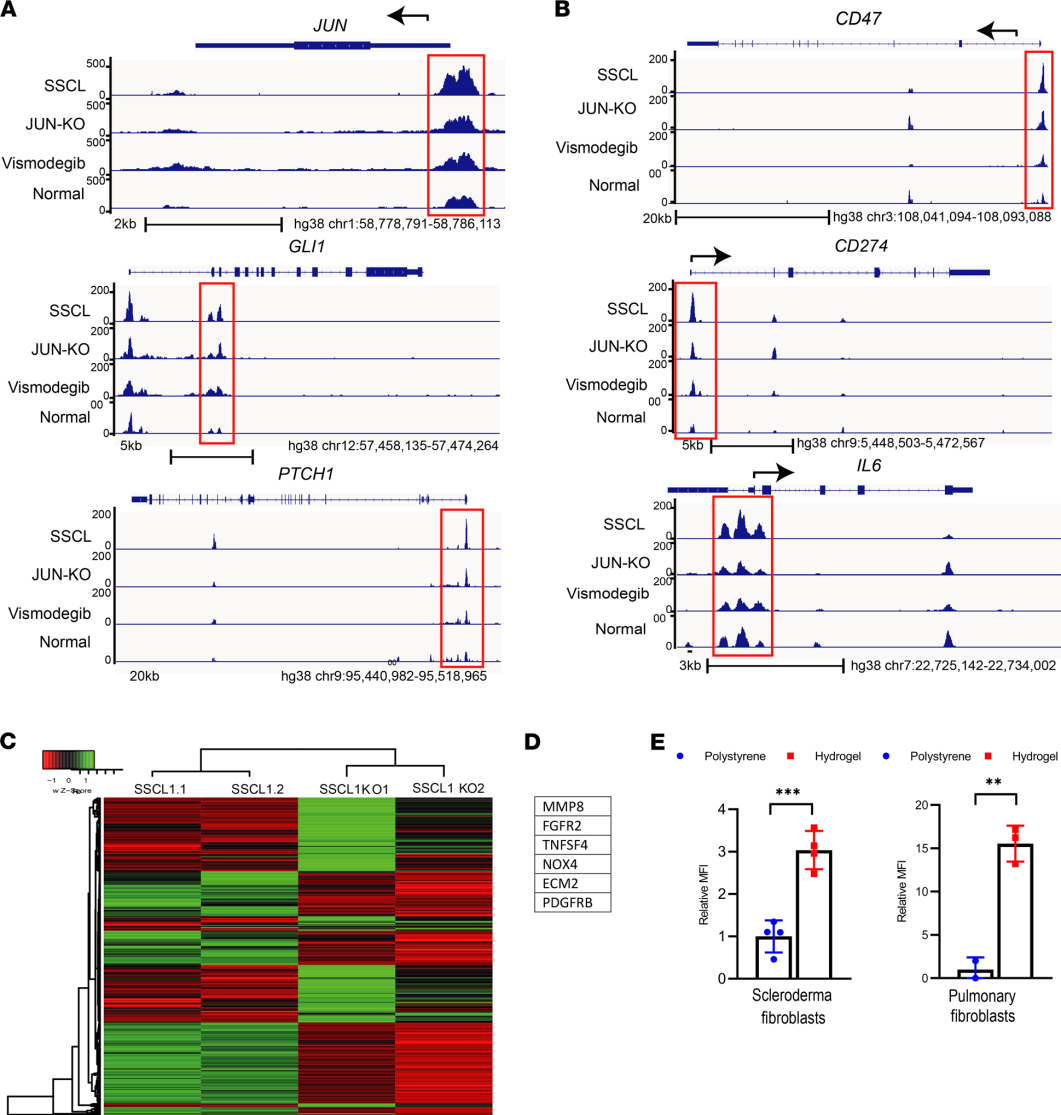
Human scleroderma increases promoter accessibilities of JUN and CD47. (**A**) ATAC-Seq analysis for JUN and the hedgehog genes *Gli1* and *Ptch1* in scleroderma fibroblasts (SSCL), scleroderma fibroblasts after JUN knockout (JUN-KO) or under vismodegib, and normal skin fibroblasts. The promoter regions are highlighted with red boxes. *n* = 2. (**B**) ATAC-Seq analysis for the immune checkpoints CD47 and PD-L1 and the interleukin IL-6 in scleroderma fibroblasts, scleroderma fibroblasts after JUN knockout or under vismodegib, and normal skin fibroblasts. The promoter regions are highlighted with red boxes. *n* = 2. (**C**) Heatmap of differential open chromatin regulatory elements characterized from ATAC-Seq. The color bar shows the relative ATAC-Seq signal (*Z* score of normalized read counts) as indicated. Samples 1 and 2 in both groups are individual samples. *n* = 2. (**D**) Fibrosis-linked genes with a 5-fold decline in promoter accessibility after JUN knockout. *n* = 2. (**E**) p-JUN expression in pulmonary fibroblasts and scleroderma fibroblasts on a 70 kPa hydrogel or a regular polystyrene plastic dish. Two-sided *t* test. **P < 0.01; ***P < 0.001. *n* = 4. Tukey’s multiple comparison test. Bars represent means with standard deviations.

At the beginning, we determined if JUN, CD47, and PD-L1 are commonly upregulated in human scleroderma. In stained tissue sections, almost all FSP1^+^ fibroblasts in scleroderma but only a minority of FSP1^+^ fibroblasts in normal skin expressed p-JUN (Figure 1, A and B). We then evaluated how primary dermal fibroblasts from scleroderma and normal skin regulate promoter accessibility of *JUN* and the hedgehog-associated genes *GLI1* and *PTCH1* through assay of transposase accessible chromatin sequencing (ATAC-Seq) studies (Figure 2A). In addition, we ran these studies after *JUN* knockout and under vismodegib to study the effects of *JUN* and hedgehog inhibition in scleroderma fibroblasts (Figure 2A). In accordance with our hypothesis, promoter accessibilities of *JUN*, *GLI1*, and *PTCH* were increased in scleroderma compared with normal skin (Figure 2A). Vice versa, knocking *JUN* out decreased the promoter accessibility of *GLI1* and *PTCH1*. As the focus of our study was to evaluate immune therapy, we analyzed the ATAC-Seq data for *CD47*, *PDL1*, and *IL6* as well (Figure 2B). Promoter accessibility of all 3 genes was increased in scleroderma fibroblasts, and *JUN* knockout led to decreased promoter accessibilities of *CD47*, *PDL1*, and *IL6*. Finally, ATAC-Seq demonstrated distinct promoter accessibility clustering before and after *JUN* knockout in scleroderma fibroblasts and increased promoter accessibilities of several fibrosis-associated genes (Figure 2, C and D). Immunostaining of human scleroderma samples confirmed the expression of CD47, CD26, and PD-1 (Supplemental Figure 1; supplemental material available online with this article; https://doi.org/10.1172/jci.insight.140458DS1). The ATAC-Seq studies did not answer which mechanism leads to the activation of JUN in scleroderma fibroblasts. One characteristic of the scleroderma skin, which it shares with all other fibrotic diseases, is its increased stiffness (20). To test if stiffer conditions themselves induce JUN activation, we plated primary scleroderma fibroblasts either on a stiff 70 kDa hydrogel or on a regular polystyrene dish. After 2 days, p-JUN was significantly increased on the hydrogel, a result that we confirmed with primary pulmonary fibroblasts (Figure 2E). In conclusion, our results with patient sections and human fibroblasts indicated that JUN was activated in human scleroderma both on the protein and on the molecular level, that JUN interacts with the hedgehog pathway, and that stiffer conditions on a hydrogel themselves induce JUN activation.

### Jun expands distinct fibroblast populations in a scleroderma model.

**Figure 3:**
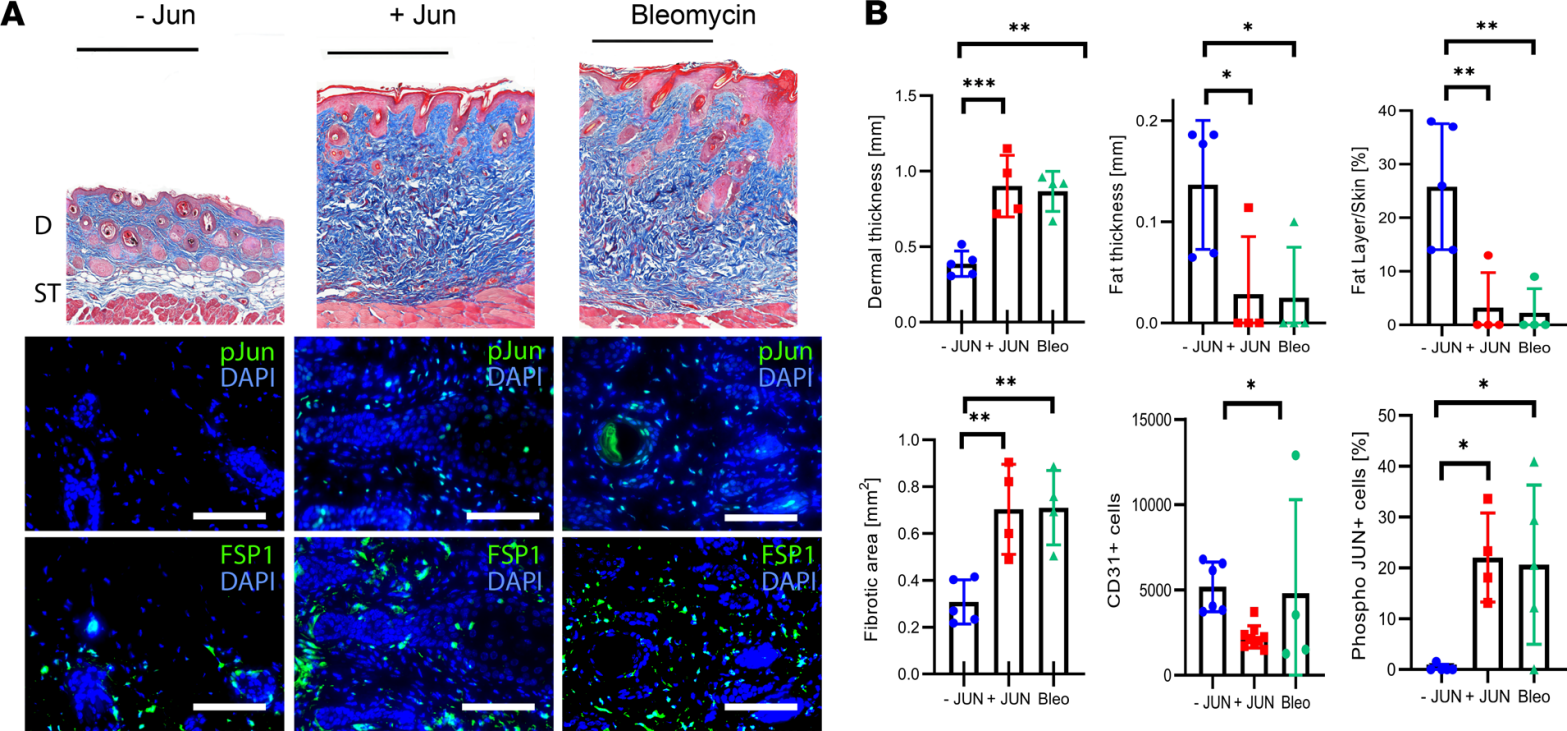
JUN induces skin fibrosis in mice. (**A**) Representative trichrome stains and immunofluorescence stains against p-JUN and FSP1 without JUN induction (-JUN), with JUN induction (+JUN), and after bleomycin injection. Black scale bar: 500 μm. White scale bar: 75 μm. D, dermis; ST, subcutaneous tissue. (**B**) Corresponding quantification of dermal thickness, fat layer thickness, fat/skin thickness relationship, fibrotic areas, total CD31^+^ endothelial cells and dermal p-JUN^+^ cells/field of view. Tukey’s multiple comparison test. *P < 0.05; **P < 0.01; ***P < 0.001. *n* = 4–8. Bars represent means with standard deviations.

**Figure 4:**
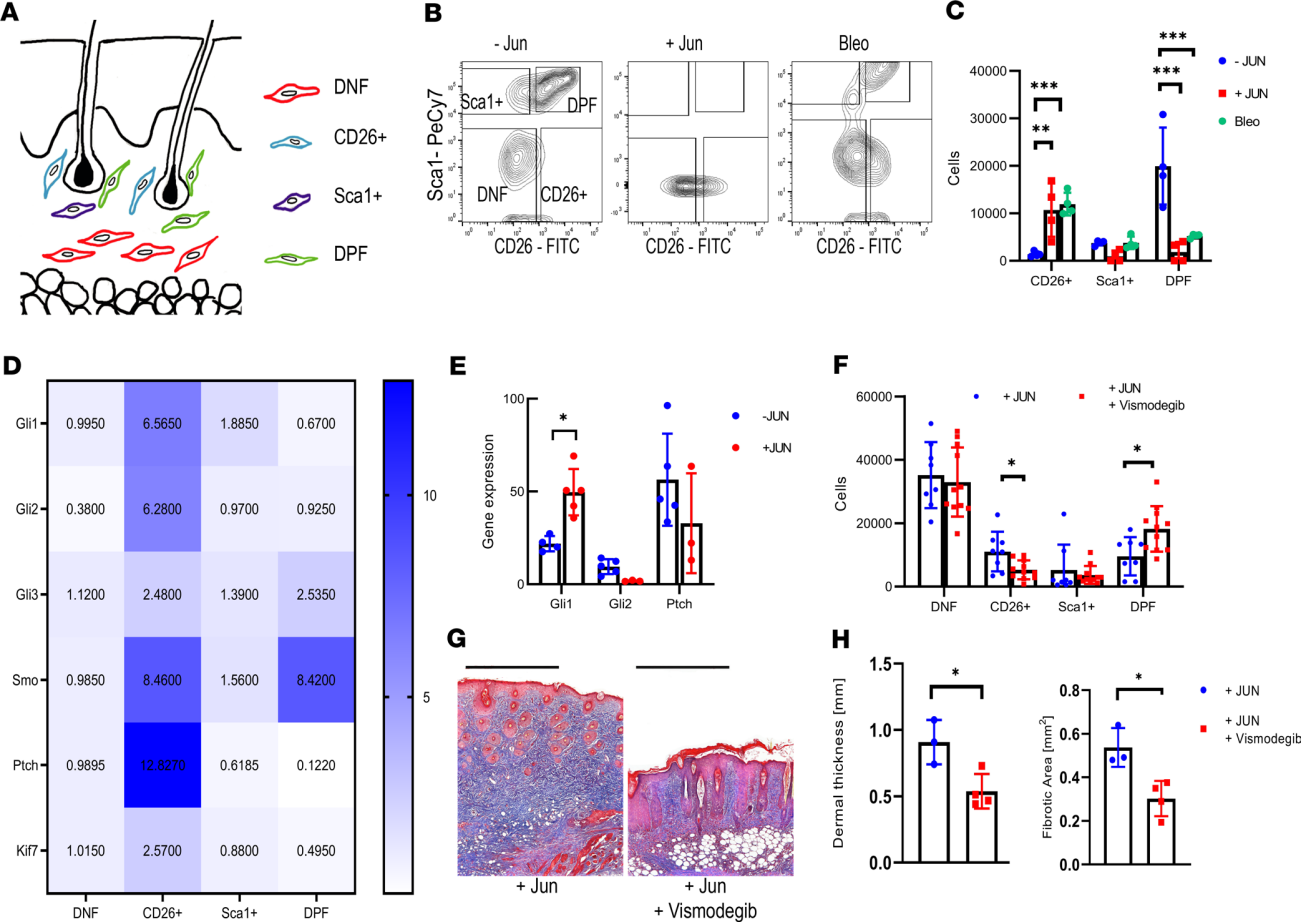
JUN expands distinct fibroblast populations in a hedgehog-dependent manner. (**A**) Schematic drawing of the localization of double-positive fibroblasts (DPF), CD26^+^ fibroblasts, Sca1^+^ fibroblasts, and double-negative fibroblasts (DNF). (**B**) Representative fibroblast FACS plots without JUN induction, with JUN induction, and after bleomycin injection. (**C**) Corresponding quantification of total CD26^+^ fibroblasts, Sca1^+^ fibroblasts, and DPF. Tukey’s multiple comparison test. **P < 0.01; ***P < 0.001. *n* = 4. Bars represent means with standard deviations. (**D**) Heatmap of the expression of different hedgehog-associated genes in the different fibroblast populations. Values are normalized to the expression in DNF. *n* = 3–6. (**E**) Expression of Gli1, Gl2, and Ptch after JUN induction. All values are compared with the same standard value. Two-sided *t* test. *P < 0.05. *n* = 3–6. Bars represent means with standard deviations. (**F**) Fibroblast populations after 3 days of Jun induction ± hedgehog inhibition. Indicated are total cells/100,000 live cells. Fisher’s multiple comparison test. *P < 0.05. *n* = 8–11. Bars represent means with standard deviations. (**G**) Representative trichrome stainings after intradermal JUN induction ± hedgehog inhibition. Scale bar: 500 μm. (**H**) Corresponding quantification of dermal thickness and fibrotic area. Two-sided *t* test. *P < 0.05. *n* = 3. Bars represent means with standard deviations.

Before studying the efficiency of CD47, we characterized the mechanisms of our *Jun*-driven mouse model and how it compared with the widely used bleomycin model (Supplemental Figure 2A) (21). We either induced *Jun* through intradermal doxycycline administrations every other day or administered intradermal bleomycin once at the beginning. After 2 weeks, both treatments led to significant fibrotic changes and a loss of fat tissue in the skin (Figure 3, A and B). Fibrotic changes included the whole dermis and the tissue below the subcutaneous muscle tissue (Supplemental Figure 2, B–D). Interestingly, both *Jun* induction and bleomycin administration induced JUN activation to the same extent (Figure 3B and Supplemental Figure 3, A and B). JUN additionally led to an increased inflammatory infiltrate (Supplemental Figure 3). We then evaluated how *Jun* and bleomycin affect distinct fibroblast populations. For this purpose, we divided CD45^–^CD31^–^CD326^–^ dermal fibroblasts into 4 groups, dependent on their expression of CD26 and SCA1 (Figure 4A, Supplemental Figure 4). Both *Jun* induction and bleomycin expanded CD26^+^SCA1^–^ fibroblasts (CD26^+^ fibroblasts) and decreased CD26^+^Sca1^+^ (double-positive, DP) fibroblasts (Figure 4, B and C). In a time course study over 5 days, *Jun* increased CD26^+^ fibroblasts gradually while it decreased DP fibroblasts directly at the beginning (Supplemental Figure 5, A–C). When *Jun* was induced longer than 5 days, CD26^+^ fibroblasts started to decrease while CD26^–^SCA1^–^ (double-negative, DN) fibroblasts expanded (Supplemental Figure 5D). As proliferation did not specifically affect CD26^+^ and CD26^–^ fibroblasts, this did suggest that immature CD26^+^ fibroblasts differentiate into mature DN fibroblasts (Supplemental Figure 5, E and F).

### CD26^+^ fibroblast expansion and skin fibrosis are hedgehog dependent.

After demonstrating that JUN causes dermal fibrosis and expands distinct CD26^+^ fibroblasts, we evaluated whether JUN distinctively influences hedgehog signaling in dermal fibroblast populations. Comparing the expression of several hedgehog-associated genes in FACS-purified dermal fibroblasts, we observed increased hedgehog activation in CD26^+^ fibroblasts, and inducing *Jun* even further increased the expression of the main hedgehog effector, *Gli1* (Figure 4, D and E; Supplemental Figure 6). To test if the hedgehog activation is mandatory for the expansion of CD26^+^ fibroblasts and the fibrotic skin changes under JUN, we blocked the hedgehog pathway with the smoothened inhibitor vismodegib (22). Hedgehog inhibition not only reduced the expansion of CD26^+^ fibroblasts but also almost completely prevented skin fibrosis after 2 weeks (Figure 4, F–H). In conclusion, these results suggested that *Jun* drives skin fibrosis through the distinct activation of hedgehog signaling in CD26^+^ fibroblasts.

### Jun mediates increased self-renewal in fibroblasts.

**Figure 5:**
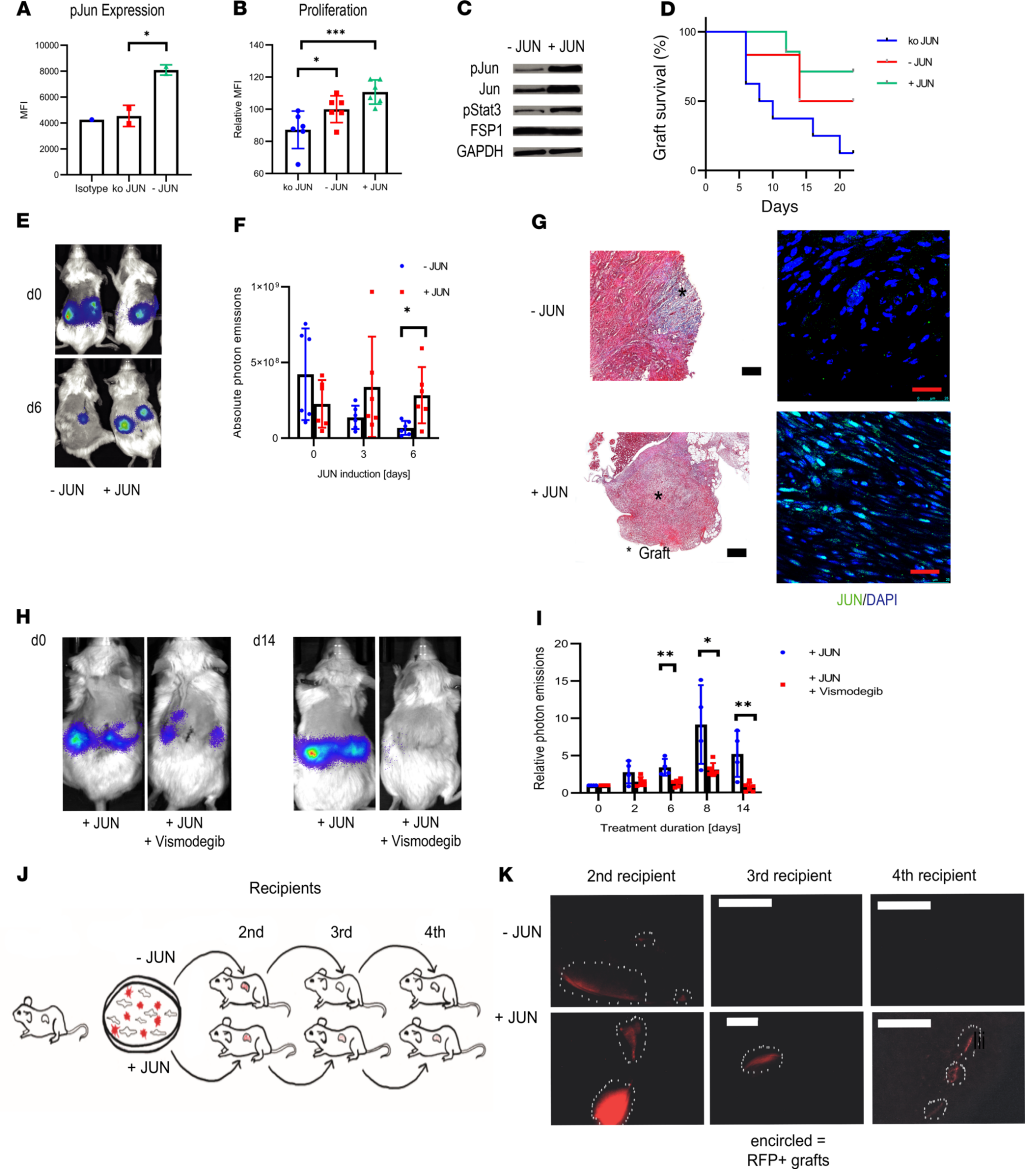
JUN mediates increased self-renewal to fibroblasts. (**A**) Mean fluorescence intensity for p-JUN after JUN knockout (ko JUN). Tukey’s multiple comparison test. *P < 0.05. *n* = 2. Bars represent means with standard deviations. (**B**) Mean fluorescence intensity for incorporated EdU (Alexa Fluor 594) after JUN knockout, without JUN induction (- JUN) and under JUN induction (+ JUN). Tukey’s multiple comparison test. *P < 0.05; ***P < 0.001. *n* = 6. Bars represent means with standard deviations. (**C**) Western blot bands for p-JUN, p-Stat3, FSP1, and GAPDH without and with JUN induction in primary mouse dermal fibroblasts. (**D**) Kaplan-Meier curve of adaptive transfer graft survivals with JUN induction, without JUN induction, and after JUN knockout. Photon emissions below 100,000 were considered as representing a lost graft. *n* = 5–8. (**E**) Representative optical images of an adaptive transfer model with JUN-inducible fibroblasts. *n* = 4–6. (**F**) Quantification of absolute photon emissions with and without JUN induction. Fisher’s multiple comparison test. *P < 0.01. *n* = 4–6. Bars represent means with standard deviations. (**G**) Corresponding trichrome (original magnification, ×10) and p-JUN stains of grafts. Black scale bar: 200 μm. Red scale bar: 25 μm. *n* = 4–6. (**H**) Representative optical images of a JUN-inducible adaptive transfer model ± vismodegib. *n* = 4–6. (**I**) Corresponding quantification of photon emissions. Values were normalized to the expression at day 0. Fisher’s multiple comparison test. *P < 0.05; **P < 0.01. *n* = 4–6. Bars represent means with standard deviations. (**J**) Schema of the adaptive serial transplantation model. (**K**) Corresponding pictures of RFP^+^ cells in the second, third, and fourth recipients with and without JUN induction. Scale bar: 500 μm. *n* = 2.

As a last step before evaluating CD47, we evaluated the effect of *Jun* on dermal fibroblasts outside their dermal niche. For this purpose, we first isolated fibroblasts from neonatal mouse skin. In vitro, an EdU uptake and subsequent flow cytometry demonstrated increased cell proliferation under *Jun* induction, and in accordance with increased cell proliferation, JUN increased p-Stat3 signaling (Figure 5, A–C; see complete unedited blots in the supplemental material). To investigate how JUN affects cell survival and proliferation in a niche-independent in vivo environment, we used an adaptive transfer model in which we transplanted GFP/luciferase-labeled primary mouse dermal fibroblasts under the kidney capsule of immunocompromised NOD/SCID-γ (NSG) mice (23). Tracking transplanted cells through luciferase-based optical imaging showed knocking *Jun* out decreased graft survival (Figure 5D). When comparing non–*Jun*-induced cells and *Jun*-induced cells, *Jun* increased cell proliferation both in optical imaging and in histology (Figure 5, E–G). Based on these and our previous results, we hypothesized that *Jun* increases self-renewal in fibroblasts. Supporting this hypothesis, blocking the stem cell–associated hedgehog pathway eliminated the fibroblast graft (Figure 5, H and I). We additionally explored a serial transplantation model in which we transplanted red fluorescence protein–labeled (RFP-labeled) fibroblasts directly from one mouse to the next (Figure 5J). While we could no longer detect any RFP-labeled cells after the second recipient without *Jun* induction, we observed RFP-labeled cells even in the fourth recipient with *Jun* induction (Figure 5K). This result strongly supports that *Jun* mediates increased self-renewal in fibroblasts.

### JUN upregulates CD47 in mouse dermal fibroblasts.

**Figure 6:**
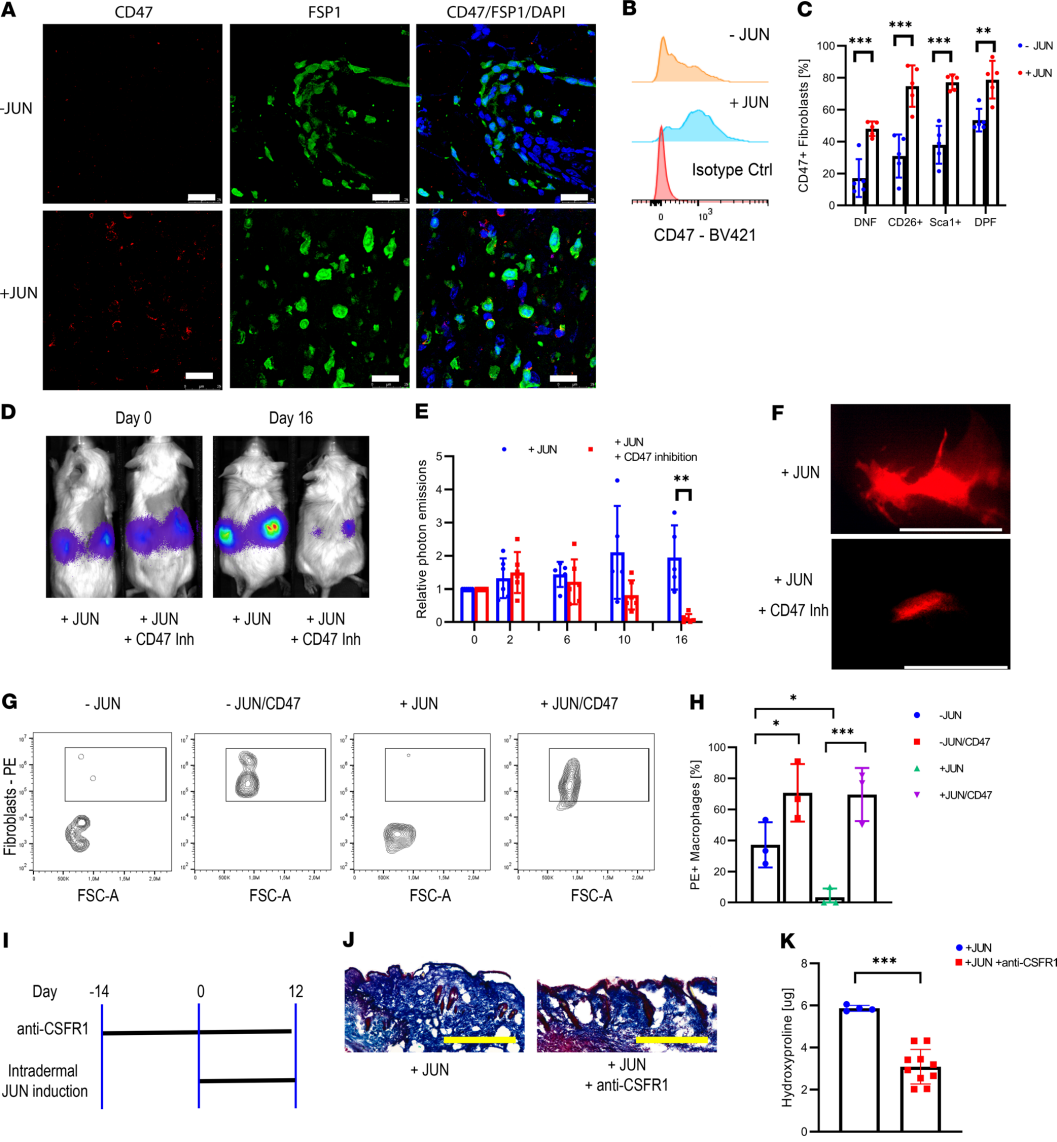
CD47 inhibition eliminates dermal fibroblasts in vivo and in vitro. (**A**) Immunofluorescence stains against CD47 and FSP1 with and without JUN induction. Scale bar: 25 μm. *n* = 5. (**B**) Histogram of CD47 expression in fibroblasts with and without JUN induction. *n* = 5. (**C**) Percentage of CD47 positivity in different fibroblast populations with and without JUN induction. Fisher’s multiple comparison test. **P < 0.01; ***P < 0.01. *n* = 5. Bars represent means with standard deviations. (**D**) Representative optical images of ectopically transplanted JUN-inducible mouse dermal fibroblasts ± CD47 inhibition. *n* = 4. (**E**) Corresponding quantification of photon emissions. Values are normalized to day 0. Fisher’s multiple comparison test. **P < 0.01. *n* = 4. Bars represent means with standard deviations. (**F**) Fluorescent graft visualization under the dissection microscope after 7 days of CD47 inhibition. Scale bar: 5 mm. *n* = 2. (**G**) FACS plot for PE/RFP^+^CD11b^+^ macrophages ± JUN induction ± CD47 inhibition in an in vitro phagocytosis assay. *n* = 3. (**H**) Corresponding quantification of RFP^+^ macrophages. Tukey’s multiple comparison test. *P < 0.05; ***P < 0.01. *n* = 3. Bars represent means with standard deviations. (**I**) Schema of a macrophage depletion trial with subsequent skin fibrosis induction. *n* = 5. (**J**) Corresponding trichrome stains. Scale bar: 500 μm. *n* = 5. (**K**) Corresponding hydroxyproline assay. Two-sided *t* test. ***P < 0.001. *n* = 5. Bars represent means with standard deviations.

Having established and characterized our *Jun*-driven mouse model, we turned to immune therapy. While *Jun* induced CD47 expression in all fibroblast subpopulations, it increased PD-L1 expression only in CD26^+^ and DP fibroblasts in vivo (Figure 6, A–C; Supplemental Figure 7, A and B). We then explored if immune therapy could eliminate primary mouse dermal fibroblasts in an adaptive transfer model. For this purpose, we treated immunocompromised mice with either a CD47 or a PD-L1 inhibitor after transplanting primary mouse dermal fibroblasts under their kidney capsule. Both inhibitors eliminated the graft (Figure 6, D–F; Supplemental Figure 7, C and D). Regarding CD47, we observed in vitro that JUN decreases and CD47 inhibition increases phagocytosis (Figure 6, G and H; Supplemental Figure 8). In regard to PD-1/PD-L1, we determined PD-1 expression on mouse macrophages, suggesting a mechanism through which PD-L1 blockade is also effective in a T cell–deficient environment, such as the immunocompromised NSG mouse (Supplemental Figure 7, E and F). Investigating the contribution of macrophages to skin fibrosis initiation showed macrophage depletion through a CSF receptor 1 (CSFR1) inhibitor ameliorated skin fibrosis (Figure 6, I–K).

### Combining CD47 and IL-6 inhibition prevents loss in subcutaneous fat tissue.

We then investigated if immune therapy targeting either the immune checkpoint PD-1/PD-L1 or the “don’t-eat-me-signal” CD47 also allows prevention of fibrosis in our *Jun*-induced mouse. Because our results had shown that both hedgehog signaling and IL-6 contributed to scleroderma, we combined both immune therapies either with vismodegib (CD47/PD-L1 inhibition + vismodegib) or with the IL-6 inhibitor tocilizumab (CD47/IL-6 inhibition). For this purpose, we concomitantly administered these treatment schedules and induced *Jun* intradermally for 2 weeks (Supplemental Figure 9A). Compared with the control group, neither treatments reduced the thickness of the skin or its fibrotic area (Supplemental Figure 9, B–D). However, the percentage of dermal fat tissue and the fatty area overall was increased with CD47/IL-6 inhibition (Supplemental Figure 9, E and F). Additionally, both treatment groups demonstrated a decreased cellular dermal infiltrate. When examining the cellular infiltrate more closely, both treatments reduced the number of CD3^+^ cells in the dermis (Supplemental Figure 9, G and H). Additionally, both treatments decreased the dermal number of KI67^+^ cells and prevented the agglomeration of macrophages (Supplemental Figure 9, G–J).

### Therapeutic CD47/IL-6 inhibition reverses skin fibrosis.

**Figure 7:**
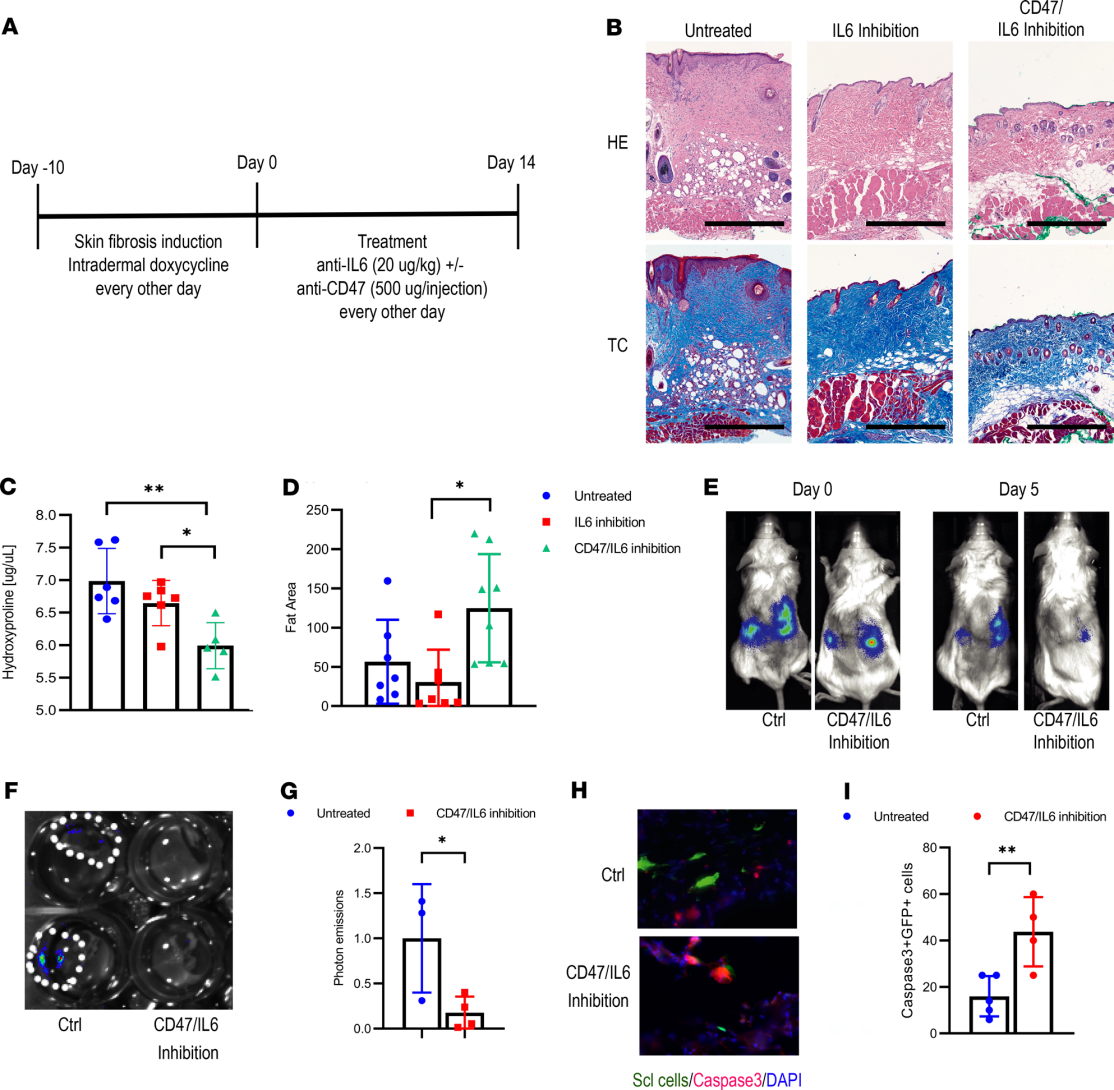
Combined CD47/IL-6 inhibition reverses skin fibrosis. (**A**) Schematic outline of the therapeutic trial. (**B**) Representative H&E and trichrome stains of the different groups. Scale bar: 500 μm. *n* = 4. (**C**) Hydroxyproline content of the skin. Tukey’s multiple comparison test. *P < 0.05; **P < 0.01. *n* = 6. Graph bars represent means with standard deviations. (**D**) Amount of fat tissue. Values indicate μm^2^/μm skin width. Tukey’s multiple comparison test. *P < 0.05. *n* = 8. Graph bars represent means with standard deviations. (**E**) Representative optical images of ectopically transplanted GFP/luciferase-labeled human scleroderma fibroblasts ± CD47/IL-6 inhibition. (**F**) Optical imaging of explanted kidneys on day 5. (**G**) Quantification of photon emissions of explanted kidney grafts normalized to the values of the untreated mice. Two-sided *t* test. *P < 0.05. *n* = 3–4. Bars represent means with standard deviations. (**H**) Corresponding caspase-3 staining of kidney grafts. Scale bar: 25 μm. (**I**) Corresponding percentage of caspase-3^+^GFP^+^ fibroblasts. Two-sided *t* test. **P < 0.01. *n* = 4–5. Bars represent means with standard deviations.

Having evaluated immune therapies in disease initiation, we then tested whether CD47 inhibition reverses skin fibrosis in mice. To study this, we induced skin fibrosis over 2 weeks before starting treatment with combined CD47/IL-6 blockade or IL-6 blockade alone (Figure 7, A and B). Encouragingly, 2 weeks after treatment initiation, the skin hydroxyproline content in the treated mice was lower than in the control groups (Figure 7, B and C). Additionally, the fat area was increased under CD47/IL-6 inhibition, reversing the skin to an almost normal state (Figure 7, B and D). Otherwise, the overall infiltrate with immune cells showed no differences between the individual treatment groups (Supplemental Figure 10, A–D). Looking for side effects, we additionally harvested other organs. Only the bone marrow demonstrated anemic changes, while the other organs all appeared normal (Supplemental Figure 10E).

### CD47/IL-6 inhibition eliminates scleroderma fibroblasts in an adaptive transfer model.

Finally, we evaluated if CD47/IL-6 inhibition accelerates the elimination of transplanted human scleroderma fibroblasts in an adaptive transfer model. Two days after transplantation, we started the treatment with CD47/IL-6 inhibition and tracked the GFP/luciferase-labeled cells through optical imaging (Figure 7E). Five days after treatment started, performing optical imaging on the explanted kidneys confirmed decreased optical signals in the treatment group (Figure 7, F and G). In accordance with the accelerated elimination of the grafts, we additionally observed significantly more apoptotic GFP^+^ fibroblasts under CD47/IL-6 (Figure 7, H and I). In summary, we were able to demonstrate that blocking CD47 and IL-6 prevented the loss in fat tissue during disease initiation, reversed fibrotic skin changes thereafter, and eliminated human scleroderma fibroblasts in an adaptive transfer model.

## Discussion

In this study, we evaluated the efficiency of the “don’t-eat-me-signal” CD47 either alone or in combination with an IL-6 inhibitor in a scleroderma mouse model. Systemic sclerosis and its skin manifestation, scleroderma, are autoimmune diseases, and current treatments mainly aim at stopping the progression of the diseases (1). A true cure with a significant reduction of fibrosis can rarely be achieved. Therefore, a therapy that allows removal of abnormal fibrosis and fibroblasts would represent a breakthrough.

We split this study into 3 parts with 3 main questions, whereby the first 2 parts cleared the way for the third part, in which we addressed the efficiency of CD47 inhibition for fibrosis. The first part served to determine whether *JUN* contributes to human scleroderma, and in the second part, we addressed mechanisms that drive skin fibrosis in our *Jun*-driven mouse model.

Regarding the role of *JUN* in human scleroderma, we demonstrate that scleroderma fibroblasts activate *JUN* on the protein level. In accordance with that, the promoter accessibility of *JUN* is increased in primary human scleroderma fibroblasts compared with normal skin fibroblasts. We additionally demonstrate that the promoters of the hedgehog-associated genes *GLI1* and *PTCH1* are more readily accessible in scleroderma fibroblasts. *JUN* knockout reduced the promoter accessibility of *PTCH1* and *GLI1*, and vice versa, the hedgehog inhibitor vismodegib reduced the promoter accessibility of *JUN*. This points toward the interconnection between *JUN* and hedgehog signaling. Importantly, the promoters of CD47 and PD-L1 were more easily accessible in scleroderma, suggesting that the abnormal fibroblasts use “don’t-eat-me-signals” and immune checkpoints as a protective mechanism against the host’s immune system. Additionally, the IL-6 promoter was more readily accessible, a finding that corresponds with a recent study in which IL-6 activated profibrotic pathways in explanted scleroderma samples (24). At the end of this part of the study, we demonstrate that stiffer conditions on a hydrogel induced JUN activation, both in scleroderma and pulmonary fibroblasts. Though this does not answer which role *JUN* plays during disease initiation, it points — in conjunction with the results from our *Jun*-driven mouse model — toward the detrimental role of *JUN* in disease progression.

In the second part of this study, we aimed at characterizing our mouse model to better interpret our results from the treatment with the CD47 inhibitor because we had to identify whether our *Jun*-driven mouse model overlaps with human scleroderma. Intradermal *Jun* induction caused dermal fibrosis and a loss in subcutaneous fat tissue. Additionally, it reduced CD31^+^ endothelial cells and increased the infiltration with CD3^+^ cells, hence exhibiting fibrosis, vasculopathy, and inflammation — 3 of 4 hallmarks of scleroderma. Except for the decrease in CD31^+^ cells, *Jun* induction mimicked the changes after bleomycin injection. Interestingly, both *Jun* induction and bleomycin led to the same extent of JUN activation. In accordance with our results from the human fibroblasts on the hydrogel, a possibility is that bleomycin first leads to a temporary fibrosis and stiffening of the skin that then leads to JUN activation. According to the *Jun*-driven mouse model, JUN activation alone is enough to cause dermal fibrosis. Once activated JUN could then maintain and worsen skin fibrosis through IL-6/p-Stat3 signaling. This mechanism could also apply to human disease, though we could not finally address this question in this study. As similar roles have been demonstrated for other AP-1 family members, such as *Fra2* and *JunB*, the deregulated activation of only 1 of the various AP-1 family members may be sufficient to promote skin fibrosis (25).

In the following experiments, we showed that JUN expanded CD26^+^ fibroblasts and decreased DP fibroblasts in the beginning. When induced over a longer time course, CD26^+^ fibroblasts then started to decrease and DN fibroblasts expanded significantly. These results suggest that during fibrosis initiation CD26^+^ fibroblasts first increased and then finally differentiated into CD26^–^ fibroblasts. In the following experiments, we demonstrated that CD26^+^ fibroblasts activated hedgehog signaling and that *Jun* induction even further increased this activation. Importantly, inhibiting the hedgehog pathway with vismodegib reversed 2 previous findings — both the expansion of CD26^+^ fibroblasts and the dermal fibrosis. In conclusion, these data demonstrate that JUN leads to dermal fibrosis through the distinct activation of hedgehog signaling in CD26^+^ fibroblasts.

There is ongoing debate about the cell from which fibrosis stems (26, 27). In an adaptive transfer model, we show that *Jun* expands dermal fibroblasts and supports graft survival independent of the dermal niche. Additionally, *Jun* induction allowed detection of transplanted fibroblasts up to the fourth recipient in a serial transplantation model, strongly indicating that JUN mediates increased self-renewal in fibroblasts. These results support the hypothesis that a clonal expansion of fibroblasts might be the source of fibrotic changes in scleroderma.

In the main part of the study, we assessed if immune therapy, targeting either CD47 or PD-L1, either in combination with vismodegib or in combination with IL-6 blockade allows us to prevent or reverse skin fibrosis. *Jun* induction increased CD47 in all and PD-L1 in 2 of the fibroblast populations. Blocking either CD47 or PD-L1 in the adaptive transfer model led to the elimination of the graft while the graft continued to grow without immune therapy. In accordance with its role as a “don’t-eat-me-signal,” CD47 inhibition increased phagocytosis in vitro. Regarding the PD-1/PD-L1 axis, we demonstrated in accordance with recent publications that mouse macrophages in our models express PD-1 as well, suggesting that PD-L1 inhibition in the T cell–deficient NSG mouse works through its interaction with PD-1 on macrophages. There is ongoing debate over the role of macrophages in fibrosis initiation. In this study, we observed that the depletion of macrophages through a CSFR1 inhibitor ameliorated skin fibrosis.

We then determined the efficiency of CD47 inhibition in the syngeneic mouse. We first demonstrated that CD47/IL-6 inhibition did not decrease the content of connective tissue in the skin in a prospective study. However, the loss of fat tissue with stiffening of the skin is a hallmark of scleroderma, and CD47/IL-6 increased the dermal fat content. Importantly, we then moved to a therapeutic study in which we first induced *Jun* over 2 weeks before starting treatment. To determine to which extent IL-6 blockade contributes to changes in skin fibrosis in our mouse model, we added an IL-6 blockade–only group. Strikingly, CD47/IL-6 decreased skin fibrosis and increased dermal fat, in comparison with both the untreated and the IL-6 blockade–only group. Exempt anemic changes, we did not observe any histological side effects under CD47/IL-6 inhibition. In accordance with these findings, CD47/IL-6 blockade accelerated the elimination of human scleroderma fibroblasts and increased apoptosis in an adaptive transfer model.

Our macrophage depletion study and our therapeutic study demonstrate that macrophages can act as a double-edged sword. On one hand they worsen fibrosis initiation; on the other hand they can remove fibrotic tissue. To protect themselves from phagocytosis, fibroblasts upregulate CD47 during disease initiation and progression, thereby enhancing their survival. Through CD47 blockade, fibroblasts become more accessible to macrophages and can finally be eliminated through phagocytosis.

Clinically, immune therapies can lead to immune-related adverse events, and safety of immune checkpoint inhibition in general, and in autoimmune disease in particular, is a primordial question (28, 29). Although data are still incomplete, retrospective studies suggest that these patients can be treated safely and effectively with immune therapies (29). Both CD47 and IL-6 inhibition are normally well tolerated. CD47 inhibition can cause anemia and infections are more common in patients treated with the IL-6 antibody tocilizumab (30, 31). However, these adverse events are rarely high grade. In accordance with that, exempt anemic changes in the bone marrow, CD47/IL-6–treated mice did not show any other histological side effects under CD47 and IL-6 inhibition.

Despite the study’s thorough evaluation of CD47 inhibition, open questions remain. One of these questions concerns the pathogenesis of scleroderma. Mouse models can only partly mimic diseases and are unsuitable to reflect the complete complexity of human disease (32). This is especially true for complex diseases such as scleroderma, whose pathogenesis is incompletely understood. There is good evidence from the literature and this study that JUN contributes to scleroderma and fibrotic diseases in general. All the same, other factors will contribute as well. A *Jun*-driven scleroderma mouse model is genetically biased and will miss contributing factors to scleroderma. Therefore, our results concerning the pathogenesis of scleroderma need to be read with caution.

Despite these limitations, our study is a proof of concept that combined immunotherapy can reverse fibrotic skin conditions in mice. The pathogenesis leading to fibrosis in different conditions may vary; however, they share an end stage with abnormal and persistent fibroblasts leading to significant morbidity and even death. Current therapies mainly stop disease progression but do not reverse fibrosis. Combining CD47 inhibition to enhance phagocytosis and IL-6 blockade to suppress underlying detrimental inflammatory processes, in contrast, could lead to a true healing of fibrosis. Regarding the good safety profiles of currently available CD47- and IL-6–blocking agents, we believe that our study gives enough evidence to safely try combined immunotherapy with CD47 and IL-6 inhibition in patients with highly fibrotic and stable, nonprogressive scleroderma.

## Methods

### Husbandry.

JUN mice were kept on a standard diet in the facilities of the Veterinary Service Center at Stanford University and had a B6/129 background. NSG mice were purchased from The Jackson Laboratory. Female and male mice were used. When different sexes were used for individual experiments, groups were sex matched. Mice were not backcrossed and were between 6 and 12 weeks of age during experiments.

### Genotyping.

To determine the genotype, we harvested tissue from the tails of newborn mice on day 10. We digested the DNA with Quickextract (Lucigen Corporation) at 68°C for 90 minutes, followed by heat inactivation at 98°C for 5 minutes. We ran the genotyping PCR for the Rosa26 and the collagen status with Phusion High Fidelity DNA Polymerase (New England BioLabs) and the same primers as described previously and indicated in Supplemental Table 1 (6).

### Adaptive transfer under the kidney capsule.

After anesthetizing mice, we shaved areas over both flanks. After we created a flank cut the subcutaneous tissue was bluntly removed from the underlying soft tissue. The abdominal wall was incised and the kidney luxated out of the abdominal cavity. After we pierced the kidney and detached the renal capsule from the renal tissue, 50,000 to 200,000 cells suspended in Matrigel were injected under the kidney capsule. Afterward, the kidney was replaced into the abdominal cavity, and the abdominal wall and the skin were separately sutured.

### Administration of vismodegib, PD-L1 inhibitor, CD47 antibody, and CSFR1 antibody.

We administered vismodegib (30 mg/kg body weight) (Selleckchem) intraperitoneally 2 times daily. The CD47 antibody (Bio X Cell) was given every other day. The first dosage was 100 μg, followed by 500 μg. For PD-L1 inhibition, we injected 100 μL (2.5 mg/mL) of high-affinity PD-1 protein anti–PD-1 daily (33). To deplete macrophages, we injected 400 μg of the CSFR1 antibody (Bio X Cell) every other day.

### Intradermal and systemic JUN induction.

To induce JUN locally, we injected 20 μL of doxycycline (MilliporeSigma) (2 mg/mL) intradermally on the back. For systemic JUN induction, we injected doxycycline intraperitoneally (20 μg/g body weight). In both systems, we performed the injections every other day.

### Luciferase-based optimal imaging.

We intraperitoneally injected 100 μL of luciferin substrate (15 mg/mL) (Biosynth). We performed optical imaging with the Lago optical imaging system (Spectral Instruments Imaging) 15 minutes later and analyzed the images with the Aura software from the same manufacturer.

### Harvesting of mouse skin for subsequent cell cultures or flow cytometry.

Mice were euthanized with CO_2_. Their backs were shaved, washed, and disinfected with 70% ethanol (EtOH). After excising the back skin, tissue was minced with scissors, followed by a digestion step in DMEM and 10% penicillin/streptomycin, supplemented with 40 μL/mL of Liberase (Roche), for 30 minutes at 37°C in the cell incubator on a shaker. The digestion reaction was quenched with DMEM and 10% FCS. The medium and tissue specimens were filtered through a 70 μm cell strainer (Falcon). Cells were then used for flow cytometry or cell culture.

### Isolation and maintenance of primary mouse dermal fibroblast cultures.

After filtering cells and tissue as described previously, cells were washed 2 times with PBS. The supernatant was removed and cell pellets and tissue parts were transferred into a culture dish. Cells were kept in DMEM and 5% human platelet lysate (HPL) supplemented with ciprofloxacin (Corning) over 5 days. Medium was changed on the first and third day. When 50% confluent, cells were split.

### Isolation of primary human scleroderma and pulmonary fibroblast cultures.

Human fibroblasts were obtained from discarded fresh lung tissues from deidentified patients. The tissue was minced and filtered through 70 μm filters, then centrifuged at 600 *g* for 5 minutes to remove nonhomogenized pieces of tissue. Tissue homogenate was treated with ACK lysing buffer (Thermo Fisher Scientific) for 10–15 minutes, centrifuged at 600 *g*, washed twice in DMEM with 10% FBS (Gibco, Thermo Fisher Scientific), and plated at a density of approximately 500,000 cells/cm^2^ in DMEM with 10% FBS, 1% penicillin/streptomycin (Thermo Fisher Scientific), and ciprofloxacin (10 μg/mL), then kept in an incubator at 37°C 95% O_2_/5% CO_2_. Medium was changed after 24 hours, and cells were cultured until 80%–90% confluent before each passage.

### Cell culture maintenance.

All cell cultures were kept in DMEM supplemented with HPL. Cells were regularly checked for signs for infection and split once they were more than 80% confluent. For splitting, cell cultures were washed with PBS and incubated with trypsin (Gibco, Thermo Fisher Scientific) for 5 minutes. The reaction was then quenched with DMEM and HPL. Cell suspensions were spun down and reapplied on cell dishes.

### Lentivirus preparation.

Around 80%–90% confluent HEK293T cells (ATCC) were transfected with 4 μg transfer plasmid (*JUN* CRISPR knockout plasmid, luciferase-GFP plasmid, or RFP plasmid), 2 μg pRRE Packing plasmid (GAG and Pol genes), 1 μg pRSV Packing plasmid (Rev gene), 1 μg pMD2.G enveloping plasmid, and 24 μg 20) polyethylenimine. The day after transfection, cell media were replaced, and cells were incubated for a further 48 hours, with media collection and replacement every 24 hours (twice). Cell media were centrifugated at 600 *g* for 10 minutes at 4°C. Then, the supernatant was filtered through a 0.22 μm strainer, ultracentrifuged at 25,000 *g* for 2 hours, aliquoted, and flash-frozen. Sequences for JUN sgRNA were forward, CACCGCCGTCCGAGAGCGGACCTTA, and reverse, AAACTAAGGTCCGCTCTCGGACGGC.

### Phagocytosis assay.

Peritoneal macrophages were harvested from non–JUN-inducible B6 mice. After euthanizing the mice, the skin above the abdomen was cut to expose the peritoneum. The abdominal cavity was then flushed with 5 mL of cold 50 mM EDTA without disrupting vessels. The injected fluid was then aspirated. After adding 10 mL of PBS, the cell suspension was centrifuged for 5 minutes at 300 *g*, followed by another washing step with PBS and a centrifugation step. The harvested cells were then plated into a 10 mL dish in regular medium. After 2 hours, the medium was exchanged and M-CSF (20 μg/mL) (MilliporeSigma) was added. After 2 days, the medium was exchanged with fresh M-CSF. In the meantime, JUN-inducible fibroblasts were prepared and labeled with an RFP plasmid. On day 2, JUN was induced in 1 group by adding doxycycline (1 μg/mL) to the cell culture medium. On day 3, macrophages and fibroblasts with and without JUN induction were harvested and counted. The fibroblast populations were then split again, 1 group with CD47 inhibition and 1 group without CD47 inhibition. Cells with CD47 inhibition were then incubated for 1 hour with the CD47 antibody (Bio X Cell) on ice in FACS buffer while the cells were kept on ice in FACS buffer as well. For measuring phagocytosis through flow cytometry, 25,000 macrophages and fibroblasts were mixed in individual wells of a 96-well plate. After a 2-hour incubation period on a shaker in a regular cell incubator, wells were washed with cold PBS, followed by trypsinization for 10 minutes. FACS buffer was added, and the plate was centrifuged, followed by another washing step with FACS buffer. After spinning down the plate, cells were incubated with a CD11b and CD45 antibody (BioLegend) for 45 minutes on ice. Afterward, cells were washed and resuspended in FACS buffer, followed by flow analysis in a CytoFLEX Flow Cytometer (Beckman Coulter). For analysis, CD45^+^CD11b^+^ cells were gated and the percentage of RFP/PE^+^ cells determined. For immunofluorescence, macrophages were plated on fibronectin-coated (MilliporeSigma) glass slides (VWR). After 45 minutes, fibroblasts were added and incubated with macrophages for 1 hour. After that, slides were vigorously washed 3 times with cold PBS, followed by a regular stain of cells plated on glass slides as described elsewhere in the Methods section.

### Hydrogel preparation.

The wells of 24-well glass-bottom plates (Mattek) were incubated with 2 M NaOH for 1 hour. After a washing step with double-distilled H_2_O (ddH_2_O), the wells were incubated with 500 μL of 2% (3-Aminopropyl)triethoxysilane (MilliporeSigma) (diluted in 95% EtOH). The wells were rinsed with ddH_2_O, followed by incubating the wells with 500 μL of 0.5% glutaraldehyde (MilliporeSigma) for 30 minutes. The wells were then rinsed with ddH_2_O and dried at 60°C. For a stiffness of 70 kPa, 46.25 μL of 40% acrylamide (MilliporeSigma) were mixed with 55.5 μL of 2% bis-acrylamide (MilliporeSigma) and 83.25 μL of ddH_2_O. Then, 1.2 μL of 10% ammonium persulfate (MilliporeSigma) and 0.8 μL of TEMED (MilliporeSigma) were added to the mixture. Each well of the plate was coated with 4 μL of the mixture and a coverslip (Glaswarenfabrik Karl Hecht). After 20 minutes, the coverslip was removed with tweezers. Next, 500 μL of 50 mM HEPES (MilliporeSigma) was added to the wells. After sterilizing the plate for 1 hour under UV light, 200 μL of 0.5% SANPAH cross-linker (ProteoChem) was added to each well. After activating the cross-linker on UV light for 10 minutes, wells were washed with 50 mM HEPES 2 times. A one-fifteenth solution of Matrigel (MilliporeSigma) diluted in 50 mM HEPES was added to the plate. The plate was incubated overnight at room temperature. Before being used, the plate was rinsed with 50 mM HEPES and incubated with regular cell culture medium for 30 minutes at 37°C in the incubator.

### Hydroxyproline assay.

We determined the hydroxyproline content with a hydroxyproline assay kit (MilliporeSigma) according to the manufacturer’s specifications. First, 10 mg of tissue was minced with scissors and homogenized in 100 μL of deionized water. Second, 100 μL of 12 M HCL was added to each sample and incubated at 120°C for 3 hours. Samples were centrifuged at 10,000 *g* for 3 minutes, and 25 μL of the supernatant was plated into a 96-well and subsequently incubated at 60°C until all liquid was evaporated. Standards and reagents were prepared as instructed in the manufacturer’s protocol. Then, 100 μL of the chloramine T oxidation buffer mixture was added to each sample and standard and incubated for 5 minutes at room temperature; 100 μL of diluted DMAB reagent was added to each sample and standard and incubated in a 60°C water bath for 90 minutes. Absorbance was read at 560 nm. All samples and standards were performed in technical duplicates.

### Preparation of HPL and HPL-containing medium.

Expired human platelets were obtained from the Stanford Blood Bank. Then, platelets were lysed through 5 quick freeze/thaw cycles. Platelet lysates were then spun down at 4000 *g* for 10 minutes, aliquoted into 15 mL tubes, and stored at –80°C. For preparing cell mediums, platelet lysates were warmed up, spun down at 4000 *g* for 10 minutes, and sequentially filtered through 0.80, 0.45, and 0.22 μm filters. The final medium, containing DMEM, 5% HPL, 1% penicillin/streptomycin, and 2 units of heparin/mL, was then filtered through a 0.22 μm filter and stored at 4°C.

### Flow cytometry and sorting.

For live cells, we washed single-cell suspensions with FACS buffer (PBS + 2% FBS + 1% penicillin/streptomycin + 1 mM EDTA + 25 mM HEPES) and then stained the cells with the primary antibodies for 45 minutes. Cells were washed and subsequently resuspended with FACS buffer. For intracellular stainings, cells were fixed with BD Wash/Perm (Becton, Dickinson and Company), followed by the same steps used for the live cells, with the exception of using BD Wash/Perm (Becton Dickinson and Company) instead of the FACS buffer. For flow cytometry, we used the CytoFlex Flow Cytometer (Beckman Coulter) for analysis only or the BD FACSAria III (Becton, Dickinson and Company) for analysis and sorting. We performed the data analysis with FlowJo (FlowJo, LLC).

### Tissue fixation and hematoxylin staining.

We kept harvested tissue in 10% formalin overnight. Tissue was then submitted to the Stanford Human Pathology/Histology Service Center for paraffin-embedding and cutting. We deparaffinized and rehydrated the tissue slides with xylene and a descending EtOH row. After washing the slides in PBS, we incubated them in hematoxylin (American MasterTech) for 4 minutes, then in bluing reagent (Thermo Fisher Scientific) for 2 minutes and in Harleco (MilliporeSigma) for 2 minutes. Slides were dehydrated with ethanol and xylene (MilliporeSigma) and mounted with Permount (Thermo Fisher Scientific).

### Trichrome staining.

We used a One Step Trichrome Stain Kit (American MasterTech). After deparaffinization and rehydration, the tissue was incubated in Bouin’s Fluid overnight, followed by Modified Mayer’s Hematoxylin for 7 minutes and One Step Trichrome Stain for 5 minutes. Slides were dehydrated with EtOH and xylene and covered with Permount (Thermo Fisher Scientific).

### Immunofluorescence staining of paraffin-embedded sections.

When using paraffin-embedded tissue, we first deparaffinized and rehydrated the tissue. Then, we performed antigen retrieval with a citric acid buffer in a pressure cooker for 15 minutes, followed by blocking with 5% serum. Sections were incubated with the primary antibody overnight at 4°C. After washing in PBS-Tween (PBST), we incubated the sections with the secondary antibody at room temperature for 30 minutes under agitation. Sections were washed, counterstained with DAPI, and mounted with Fluoromount-G (SouthernBiotech). Images of histological slides were obtained on a Nikon Eclipse E400 microscope equipped with a SPOT RT color digital camera model 2.1.1 (Diagnostic Instruments).

### Immunofluorescence staining of OCT-embedded sections.

For OCT sections, harvested tissue was fixed in 4% PFA for at least 2 hours, followed by an incubation in 30% sucrose overnight. Tissue was then embedded in OCT and stored at –80°C. Thereafter, the OCT block was cut into 10 μm sections. Sections were stored at –20°C. Before staining, sections were dried for at least 15 minutes at room temperature. Sections were rehydrated in PBS. For intranuclear stains, sections were incubated for 10 minutes in Triton X-100 at room temperature. Otherwise, cells were blocked with 5% for 1 hour. The remaining steps were the same as for the immunofluorescence stains of paraffin-embedded tissue.

### Immunofluorescence staining of cells plated on glass slides.

For plating cells on glass slides (VWR), areas were circled on glass slides with a fat pen (Vector Laboratories), followed by sterilization for at least 1 hour under UV light. Then, fibronectin (20 μg/mL PBS) was added. After incubating the slides for 1 hour in a cell incubator at 37°C, the fibronectin solution was aspirated, and the slides were dried in the cell culture cabinet. Cells were added onto the glass slides and incubated for different amounts of times in regular cell culture medium. Thereafter, the glass slides were washed in PBS, followed by fixing the cells with 4% paraformaldehyde at room temperature for 10 minutes. The slides were washed again in PBS, followed by permeabilization in Triton X-100 for 10 minutes for intranuclear staining. Otherwise, cells were blocked with 5% serum for 1 hour. Afterward, the staining procedure matched the staining protocol of the paraffin-embedded slides.

### Immunohistochemistry.

When using paraffin-embedded tissue, we first deparaffinized and rehydrated the tissue. Then, we performed antigen retrieval with a citric acid buffer in a pressure cooker for 15 minutes, followed by blocking with 5% serum. Sections were incubated with the primary antibody overnight at 4°C. After washing in PBST, we incubated the sections with the secondary HRP antibody at room temperature for 30 minutes under agitation. Sections were washed in PBST and ddH_2_O followed by incubation for 15–20 minutes in AEC Peroxidase Substrate (Vector Laboratories). Sections were washed in ddH_2_O and incubated in Modified Mayer’s Hematoxylin for 4 minutes. After washing in ddH_2_O, slides were mounted with Fluoromount-G (SouthernBiotech).

### RNA extraction, cDNA, and quantitative PCR.

FACS-purified cells were sorted into TRIzol (Thermo Fisher Scientific). For RNA extraction, we added chloroform and centrifuged the tubes. We transferred the upper phase to a new tube and added 70% EtOH. Afterward, the complete volume was added to the columns of the RNeasy MinElute Cleanup Kit (QIAGEN). Between the next 3 centrifugation steps, we washed the columns with 80% EtOH (+H_2_O), 80% EtOH (+RPE), and 70% EtOH. After letting the membranes slightly dry, we added water onto the membranes, centrifuged the columns, and measured the RNA quantity and quality of the flow through with a NanoDrop 2000 (Thermo Fisher Scientific). For cDNA creation, we used the iScript Advanced cDNA Synthesis Kit (Bio-Rad) according to the manufacturer’s specifications. Up to 1 μg of RNA was transformed into cDNA. We then ran quantitative PCR on a 7900 HT Fast-Time PCR System (Applied Biosystems, Thermo Fisher Scientific). Reactions contained 2.5 μL of H_2_O, 5 μL of PowerUp SYBR Green Master Mix (Applied Biosystems, Thermo Fisher Scientific), 1 μM of primers (0.5 μL), and 2 μL of cDNA.

### Western blotting.

Cells in a well of a 6-well plate were lysed with 150–200 μL of urea buffer. Cell lysates were sonicated and centrifuged. Protein concentrations were determined with the Pierce BSA Protein Assay (Thermo Fisher Scientific). Then, 10 μg of protein was mixed with loading dye and heated up to 99°C for 5 minutes. Protein lysates were added to a Bolt 4-12% Bis-Tris-Plus Gel (Invitrogen, Thermo Fisher Scientific). After 30 minutes, proteins were transferred to a nitrocellulose membrane for 90 minutes. Membranes were blocked with 5% milk powder in PBST for 30 minutes, followed by incubating the membranes with the primary antibody diluted in the blocking buffer for 1 hour at room temperature. Membranes were washed 3 times with PBST for 5 minutes each. Membranes were incubated with the secondary antibody diluted in the blocking buffer at room temperature, followed by 3 washing steps in PBST. Membranes were incubated with Luminata Forte Western HRP Substrate (MilliporeSigma) for 5 minutes and developed on UltraCruz Autoradiography films (Santa Cruz Biotechnology). Membranes were stripped with Restore Western Blot Stripping Buffer (Thermo Fisher Scientific) for 5 minutes. Membranes were then blocked, incubated with primary and secondary antibodies, and developed as described in this section.

### Primary antibodies.

For flow cytometry we used CD3 (BioLegend, 100209, clone 17A2), CD4 (BioLegend, 100422, clone GK1.5), CD11b (BD, 554411, clone M1/70), CD11c (BioLegend, 117324, clone N418), CD25 (BioLegend 102035, clone PC61), CD26 (BioLegend, 137805, clone H194-112), CD31 (BD, 553373, clone MEC 13.3), CD45 (BioLegend, 103110, clone 30-F11), CD47 (BioLegend, 127527, clone Miap301), CD326 (BioLegend, 118218, clone G8.8), F4/80 (BioLegend, 123116, clone BM8), PD-L1 (BioLegend, 124312, clone 10F.9G2), Sca1 (BioLegend, 108114), and phospho–c-Jun (Ser73) (Cell Signaling Technology [CST], 32705, clone D47G9). For immunohistochemistry/immunofluorescence we used adiponectin (Abcam, ab22554), CD3 (Abcam, ab5690), CD11b (Novus, NB110-89474), CD26 (Abcam, ab28340), CD26 (R&D Systems, Bio-Techne, AF954), CD31 (Dako, m0823), CD47 (Thermo Fisher Scientific, 14-0479-82, clone B6H12), CD47 (R&D Systems, Bio-Techne, AF1866), CD68 (Agilent, GA60691-2, clone KP1), cleaved caspase-3 (CST, 96645, clone 5A1E), FSP1 (MilliporeSigma, 07-2274), FSP1 (Abcam, ab58597), collagen 1 (Abcam, ab34710), FSP1 (MilliporeSigma, 07-2274), Ki67 (Abcam, ab15580), PD-1 (Cell Marque, 315M-96, clone NAT105), PD-1 (R&D Systems, Bio-Techne, AF1021), PD-L1 (R&D Systems, Bio-Techne, AF1019), and phospho–c-Jun (Ser73) (CST, 32705, clone D47G9). For Western blot we used c-Jun (CST, 9165S, clone 60A8), FSP1 (MilliporeSigma, 07-2274), GAPDH (GeneTex, 627408, clone GT239), phospho–c-Jun (Ser73) (CST, 32705, clone D47G9), and phospho-Stat3 (CST, 9131S).

### Secondary antibodies.

Secondary antibodies were Alexa Fluor (AF) 488 goat anti-rabbit (CST, 44125), AF 594 goat anti-rabbit (Invitrogen, Thermo Fisher Scientific, A-11012), AF 594 donkey anti-goat (Novus, NBP1-75607), AF 594 goat anti-mouse (Invitrogen, Thermo Fisher Scientific, A-11032), and HRP goat anti-rabbit (Abcam, 205718).

### ATAC-Seq library preparation, sequencing, and data preprocessing.

The ATAC-Seq was performed as described before (34). Briefly, 50,000 cells were collected and washed with cold PBS and lysed using 0.1% NP-40 in resuspension buffer. Tn5 transposition of nuclei pellets was carried out at 37°C for 30 minutes, using the DNA sample preparation kit from Nextera (Illumina). The reaction was purified using QIAGEN MinElute columns and then amplified for 8–15 cycles to produce libraries for sequencing. ATAC-Seq libraries were sequenced on Illumina HiSeq 4000. ATAC-Seq paired-end reads were trimmed for Illumina adapter sequences and transposase sequences using Kundaje ATAC_pipelines. The libraries were initially sequenced on a MiSeq sequencer (Illumina) and analyzed using a custom script to determine the enrichment score; only libraries that had the highest score above the threshold (>5) were chosen for deeper sequencing. Two independent biological replicates were sequenced per sample. The data have been uploaded to the NCBI’s Gene Expression Omnibus under the accession number GSE151943.

### Deep sequencing data analysis.

Differentially accessible peaks from the merged union peak list were selected with the DESeq2 package from bioconductor using raw read counts of each sample using log_2_ fold change > 1 and *P* < 0.05. The read counts of the differential peaks in each sample were further normalized by *Z* score transformation. Peak genomic annotation was performed by HOMER package. Hierarchical clustering was used to cluster the peaks and samples. The results were presented as a heatmap by Morpheus from the Broad Institute.

### Statistics.

We used GraphPad Prism (GraphPad Software Inc) for creating graphs and running statistical analyses. When 2 values were compared, a 2-sided *t* test was used, if more than 2 values were analyzed. When more than 2 values were directly compared with each other, we used the Tukey’s multiple comparison test. When 2 values were compared over different time points, without comparing the time points to each other, we used Fisher’s multiple comparison test. Generally, experiments included at least 3 independent values from 2 independent experiments. *P* values below 0.05 were considered statistically significant. Regarding naturally occurring higher variation in animal trials, we determined before the experiment to exclude the highest and lowest values (when *n* was at least 8) or the 2 highest and 2 lowest values (when *n* of 16 was reached).

### Study approval.

Animal trials were approved by the Stanford Administrative Panel on Laboratory Animal Care (approval 30911). Tissue for human primary cultures was obtained from discarded fresh skin specimens from deidentified patients.

## Author contributions

TL conceived and interpreted the study, ran and analyzed all experiments except ATAC-Seq studies and Western blots, and wrote the manuscript. L Cui was responsible for running and analyzing ATAC-Seq studies. MEK participated in running and analyzing animal trials shown in Figures 6–8 and writing the manuscript. TC ran all Western blots. CM wrote the manuscript. L Chung, RB, and KR provided materials. TS wrote the manuscript. GW conceived the study, interpreted data, and gave final manuscript approval.

## Supplementary Material

Supplemental data

## Version 1 08/20/2020

Electronic publication
