# Supplementary material for: CD47 prevents the elimination of diseased fibroblasts in scleroderma
Source: JCI Insight. 2020 Aug 20;5(16):e140458. doi: 10.1172/jci.insight.140458 (PMC7455137; doi:10.1172/jci.insight.140458)
Supplement: Supplemental data [file jciinsight-5-140458-s141.pdf]

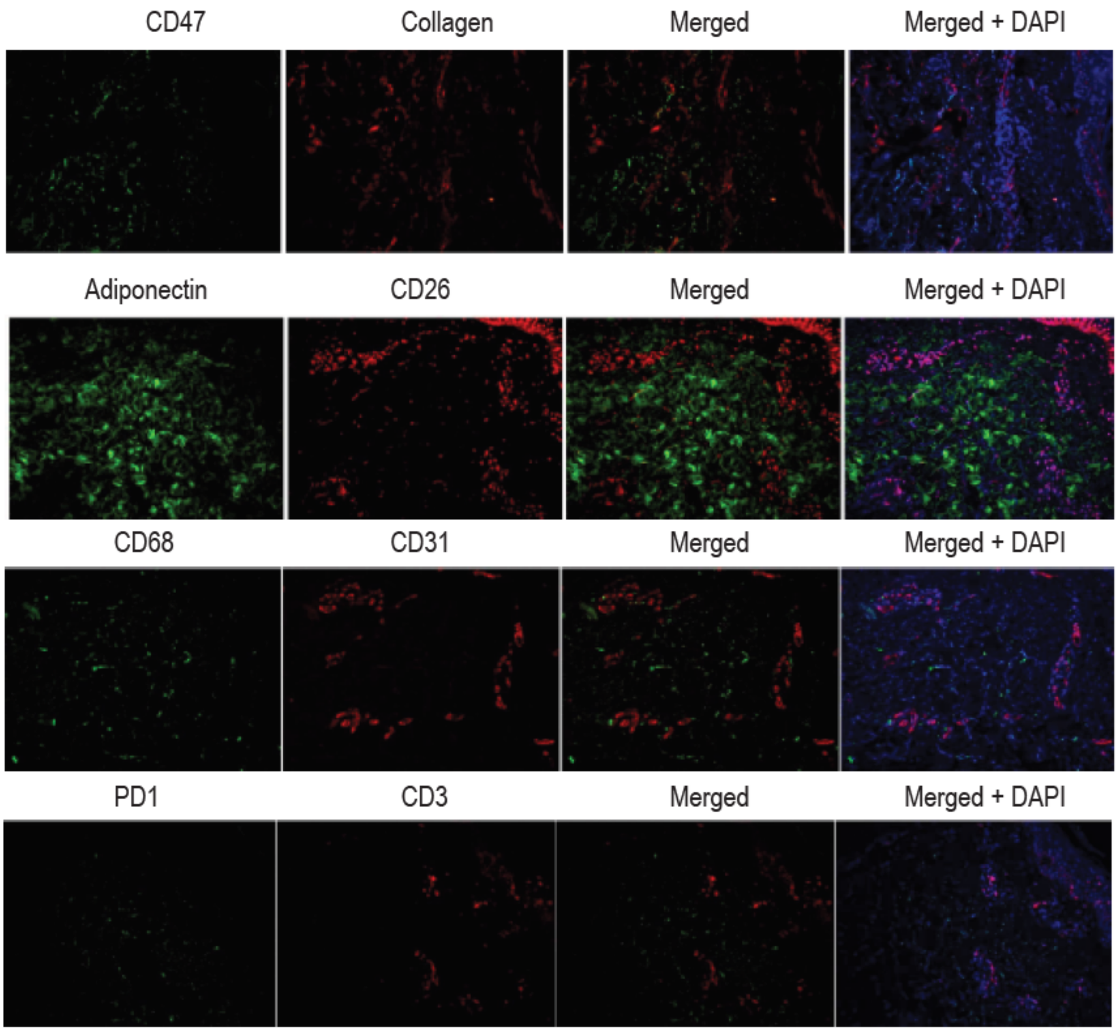

Supplementary Figure 1. Expression of different markers in human scleroderma samples. Chromatin accessibility changes in human scleroderma samples.

Representative images of human scleroderma skin sections stained against different markers.

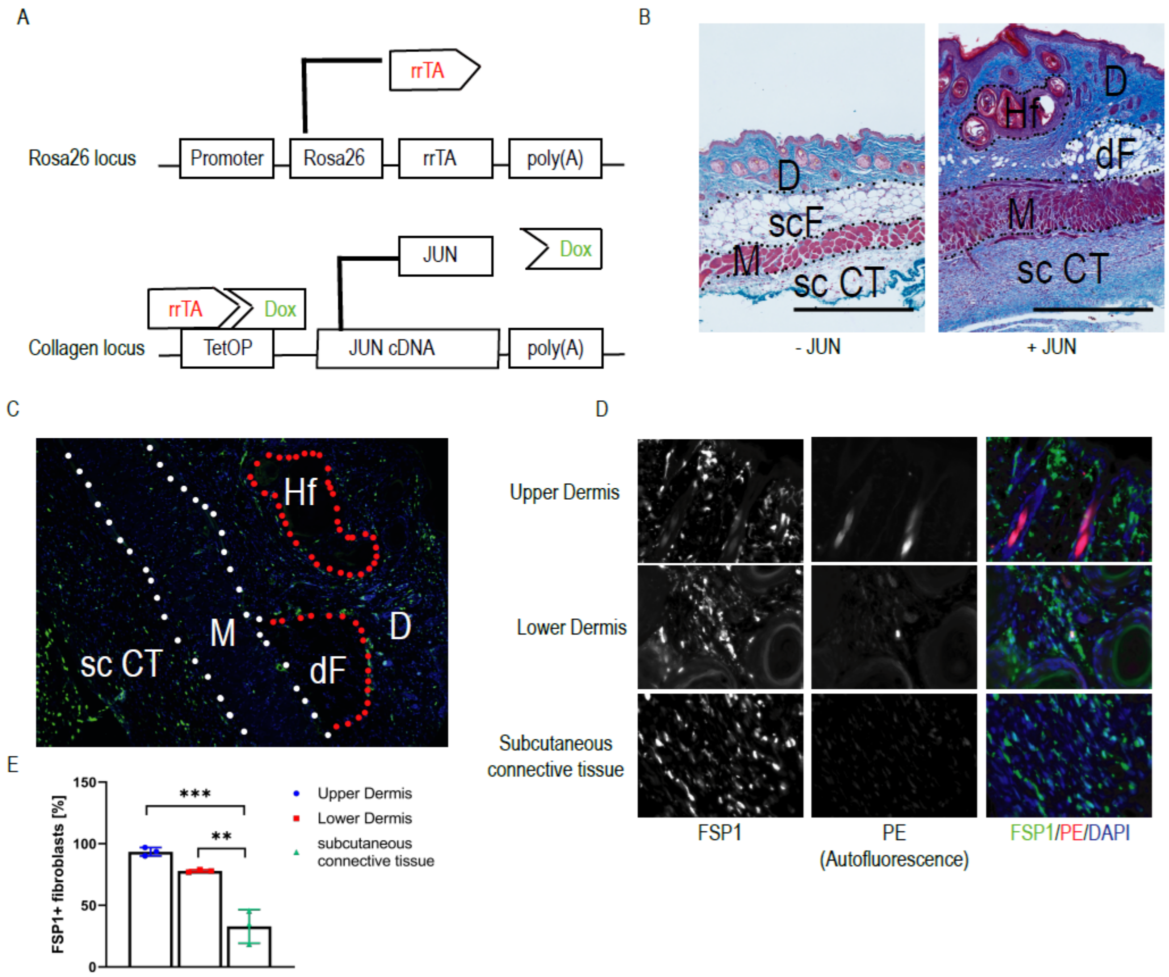

Supplementary Figure 2. Jun expands FSP1+ fibroblasts throughout the skin and the underlying tissue.

(A) Genetic modifications of the JUN-inducible mouse. rrTA = reverse tetracycline transactivator, TetOP = Tetracycline/doxycycline-responsive operator

(B) Representative Trichrome-stained whole skin sections without (-JUN) and with JUN induction (+JUN). D = Dermis, scF = subcutaneous fat, M = subcutaneous muscle, sc CT = subcutaneous connective tissue, dF = dermal fat, Hf = Hairfollicle. Scale bar = 500 µm.

(C) Whole skin section after Jun induction (corresponding to the section B). Green = FSP1+, blue = DAPI. D = Dermis, M = subcutaneous muscle, sc CT = subcutaneous connective tissue, dF = dermal fat, Hf = Hairfollicle

(D) Quantification of FSP1+ fibroblasts in the upper dermis, the lower dermis and the subcutaneous connective tissue. Indicated are the percentages of FSP1+ cells among all spindle-shaped fibroblasts. Turkey's multiple comparisons test. ** $p < 0.01$ *** $p < 0.001$. Scale = 500 µm. n=3. Bar graphs represent means with standard deviations.

(E) Representative stains against FSP1 in the upper and lower dermis and the subcutaneous connective tissue after Jun induction.

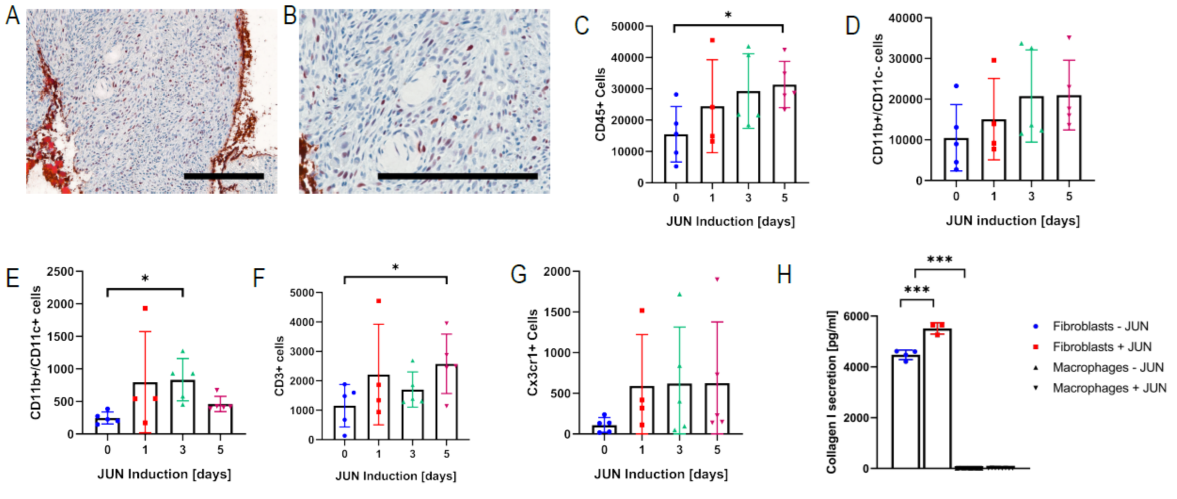

Supplementary Figure 3. Skin immune filtration under JUN induction.

(A) Representative IHC stain against pJUN. Scale bar = 200 µm.

(B) Representative IHC stain against pJUN. Scale bar = 200 µm.

(C) Quantification of CD45+ cells under JUN induction over up to 5 days. Turkey's multiple comparisons test. * $p < 0.05$. n=4-5. Bar graphs represent means with standard deviations.

(D) Quantification of myeloid CD11b+ cells under JUN induction over up to 5 days. Fisher's multiple comparisons test.. n=4-5. Bar graphs represent means with standard deviations.

(E) Quantification of dendritic CD11b+CD11c+ cells under JUN induction over up to 5 days. Turkey's multiple comparions test. * $p < 0.05$. n=4-5. Bar graphs represent means with standard deviations.

(F) Quantification of CD3+ T cells under JUN induction over up to 5 days. Turkey's multiple comparisons test. * $p < 0.05$. n=4-5. Graph bars represent means with standard deviations.

(G) Quantification of hematopoietic Cx3cr1+ cells under JUN induction over up to 5 days. Fisher's multiple comparisons test. n=4-5. Graph bars represent means with standard deviations.

(H) In vitro collagen 1 secretion of fibroblasts and macrophages +/- JUN induction. One-way ANVOA * $p < 0.05$ *** $p < 0.001$ (n=4). Bar graphs represent means with standard deviations.

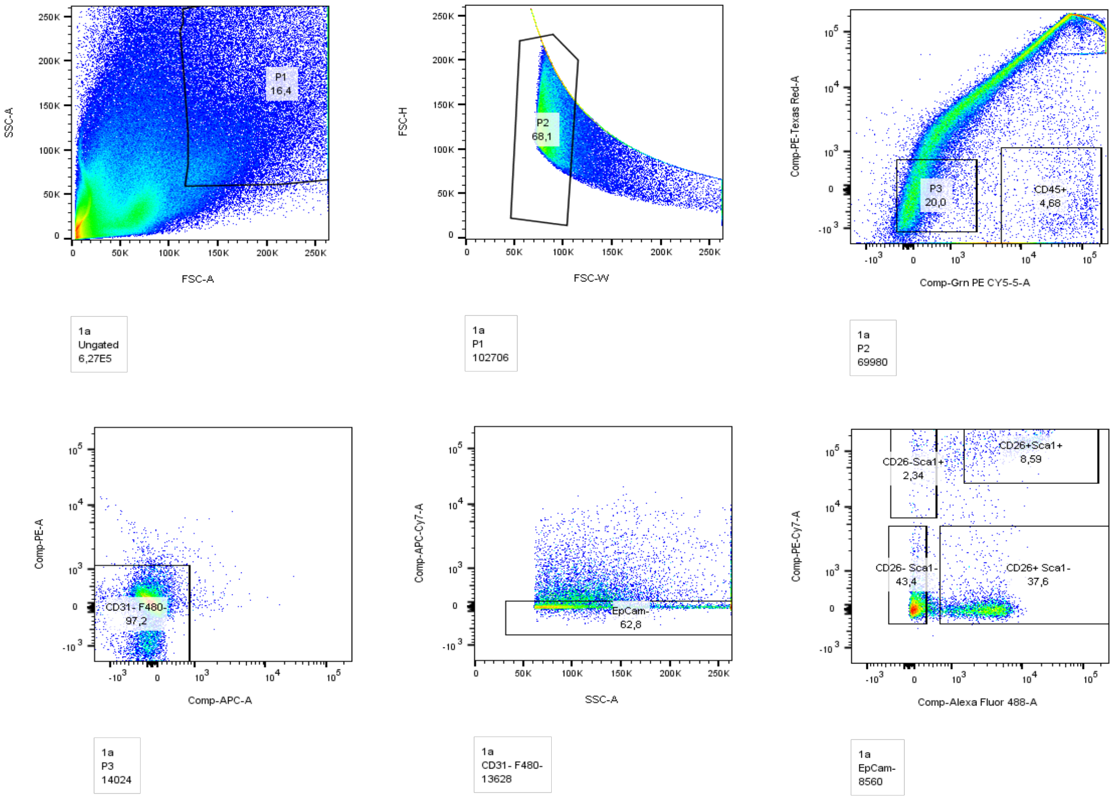

Supplementary Figure 4. Fibroblast gating strategy.

After identifying cells and then single cells, hematopoietic (CD45+) and dead cells (PI) are excluded. In a next step, macrophages (F4/80+) and endothelial (CD31+) cells are excluded. After removing epithelial (CD326+) cells, fibroblasts are divided, based on their expression of CD26 and Sca1.

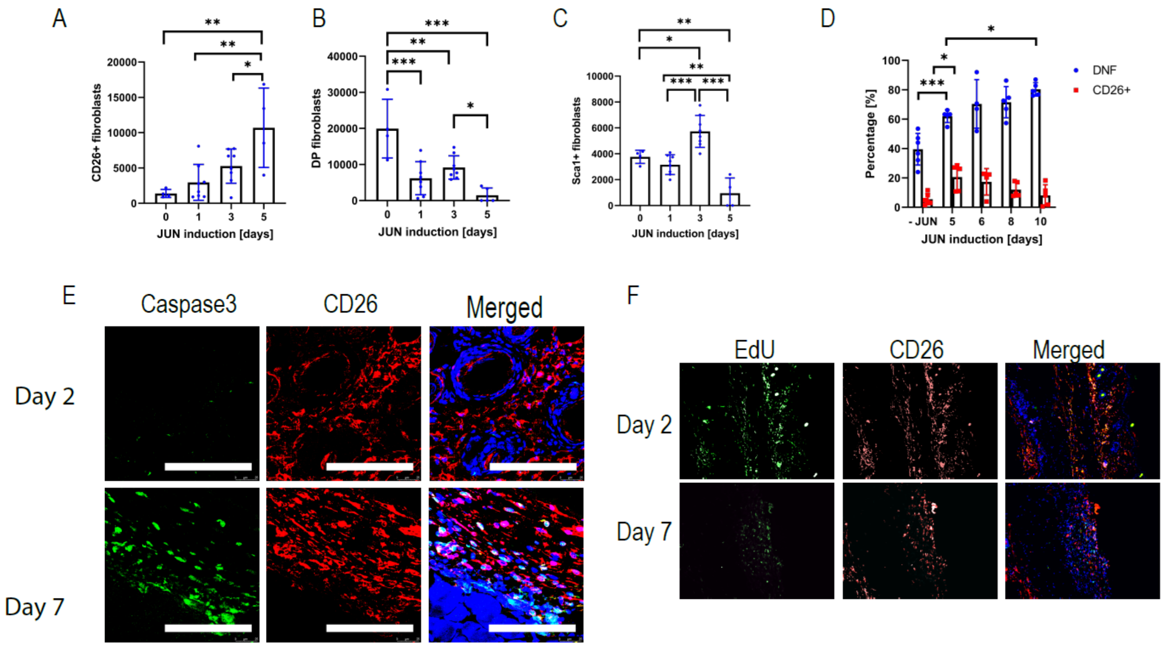

Supplementary Figure 5. JUN initially expands CD26+ fibroblasts.

(A) Quantification of CD26+ fibroblasts, over five days of JUN induction. Turkey's multiple comparisons test. * $p < 0.05$ ** $p < 0.01$. n=4-5. Bar graphs represent means with standard deviations.

(B) Quantification of DPF over five days of JUN induction. Turkey's multiple comparisons test. *** $p < 0.001$. n=5. Bar graphs represent means with standard deviations.

(C) Quantification of Sca1+ fibroblasts over five days of JUN induction. Turkey's multiple comparisons test. ** $p < 0.01$ *** $p < 0.001$. n=5. Bar graphs represent means with standard deviations.

(D) Quantification of CD26+ fibroblasts and DP fibroblasts (DPF) over up to 10 days of JUN induction. Fisher's multiple comparisons test. * $p < 0.05$ ** $p < 0.01$ *** $p < 0.001$. n=4-6. Bar graphs represent means with standard deviations.

(E) Representative immunofluorescence stains against Caspase3 and CD26 two and seven days after JUN induction. Scale bar = 100 µm. **(F)**

Representative immunofluorescence stain against CD26 and corresponding EdU visualization two and seven days after JUN induction.

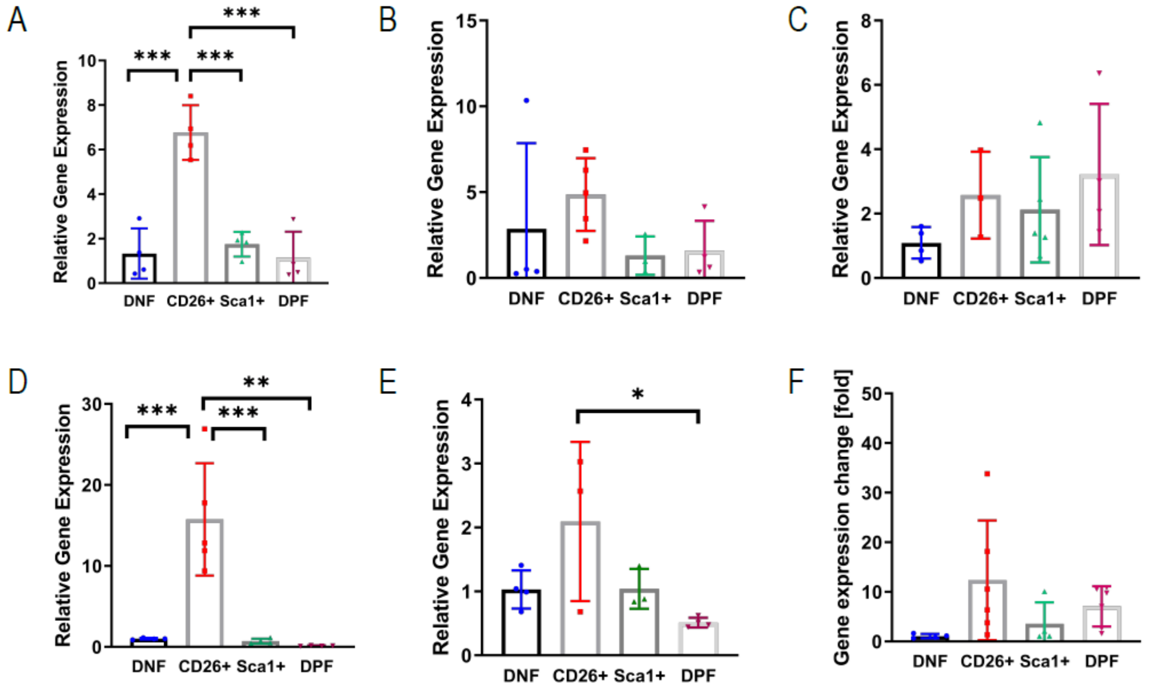

Supplementary Figure 6. CD26+ fibroblasts activate hedgehog signaling.

Normalized qPCR data from facs purified fibroblast populations from non JUN-induced JUN mice. The values for each gene are compared to the mean value of the DN fibroblasts.

(A) Gli1. Turkey's multiple comparisons test. *** $p < 0.001$. n =4. Bar graphs represent means with standard deviations.

(B) Gli2. Turkey's multiple comparisons test. n=3-5. Bar graphs represent means with standard deviations.

(C) Gli3. Turkey's multiple comparisons test. n=3-5. Bar graphs represent means with standard deviations.

(D) Ptch1. Turkey's multiple comparisons test. ** $p < 0.01$ *** $p <0.001$. n=3-5. Bar graphs represent means with standard deviations.

(E) Kif7. Turkey's multiple comparisons test. * $p < 0.05$. n=3-4. Bar graphs represent means with standard deviations.

(F) Smo. Turkey's multiple comparisons test. n=4-6. Bar graphs represent means with standard deviations.

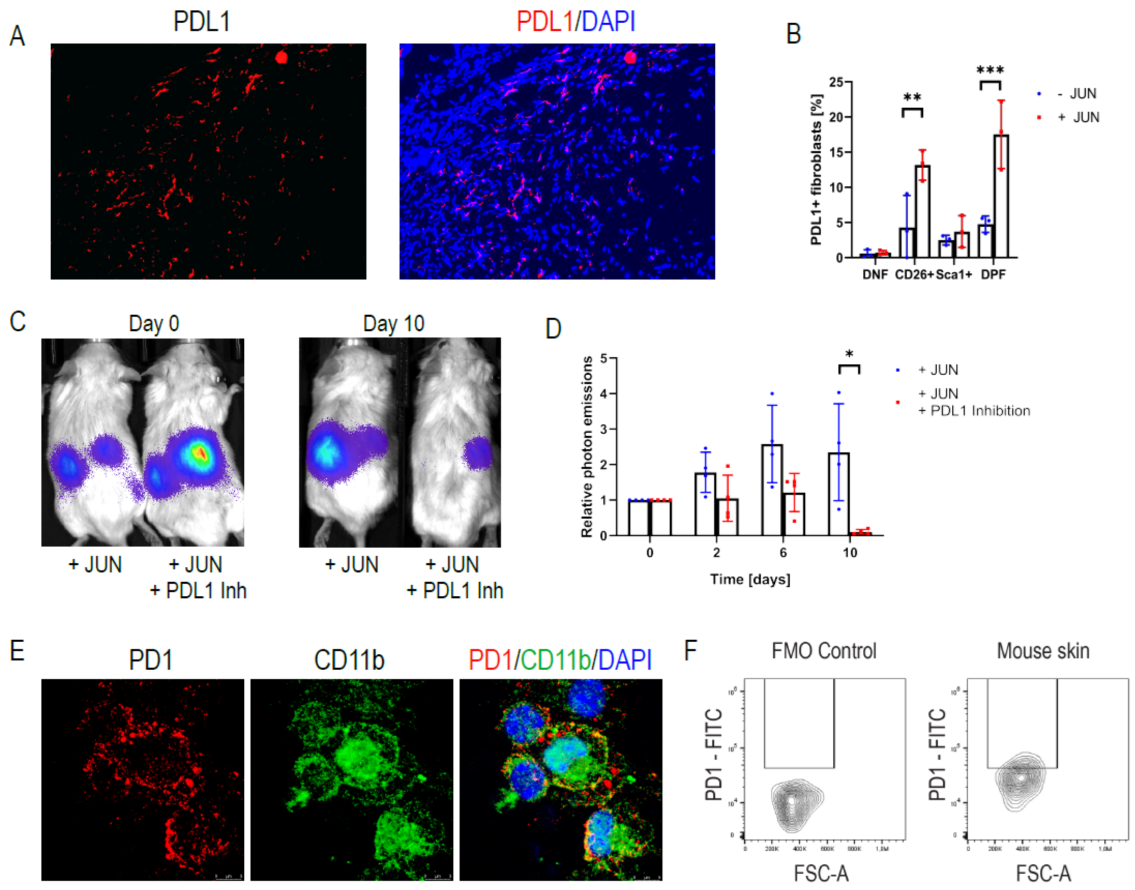

Supplementary Figure 7. PDL1 inhibition eliminates ectopic fibroblasts.

(A) Immunofluorescence stains against PDL1 after local JUN induction in skin after 14 days

(B) PDL1 expression in different subsets of fibroblasts with and without JUN induction. Turkey's multiple comparisons test. ** $p < 0.01$ *** $p < 0.001$. n=3. Bar graphs represent means with standard deviations.

(C) Representative optical images of ectopically transplanted JUN inducible fibroblasts +/- PDL1 inhibition. n=4. **(D)** Corresponding quantification of photon emissions. Turkey's multiple comparisons test. * $p < 0.05$. n=4. Bar graphs represent means with standard deviations.

(E) Immunofluorescence stains against PD1 and CD11b on macrophages harvested from the peritoneum.

(F) FACS plots of PD1 expression in CD45+CD11b+ blood cells. n=2. Graph bars represent means with standard deviations.

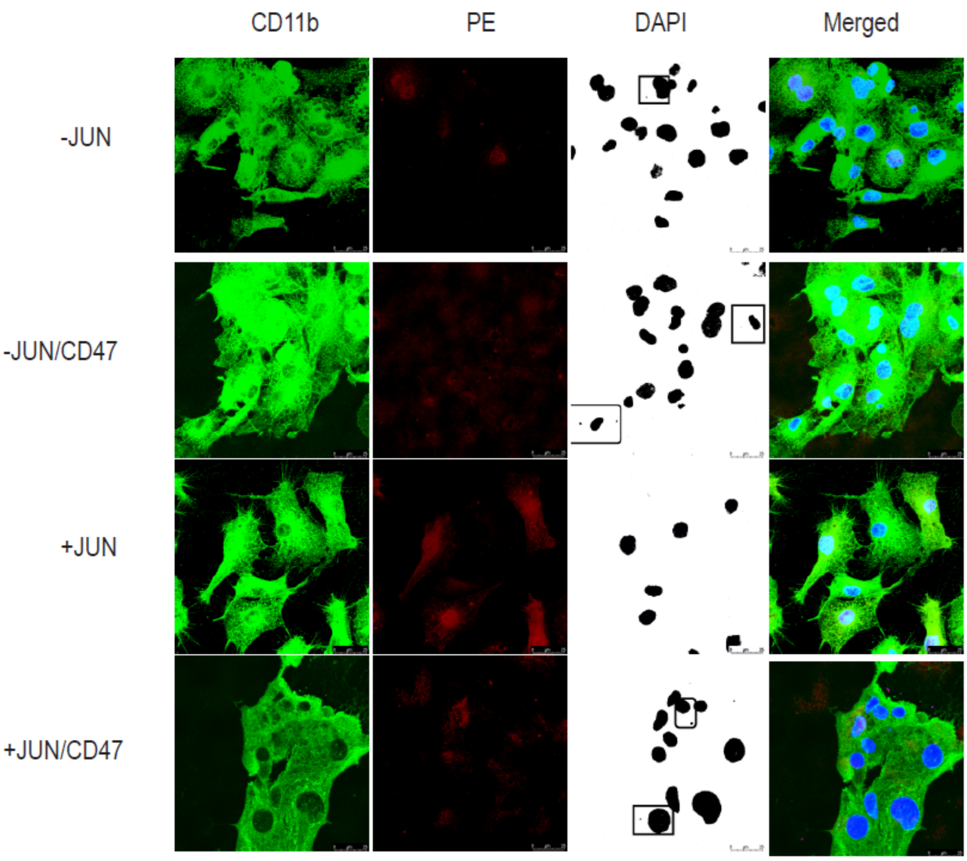

Supplementary Figure 8. CD47 inhibition increases phagocytosis of dermal fibroblasts in vitro.

Images are taken with a confocal microscope. RFP+ target cells are detected in the PE channel. Macrophages who have fully digested target cells are marked by small isolated DNA pieces. Boxes in the DAPI represent macrophages with additional DNA pieces as signs of advanced phagocytosis.

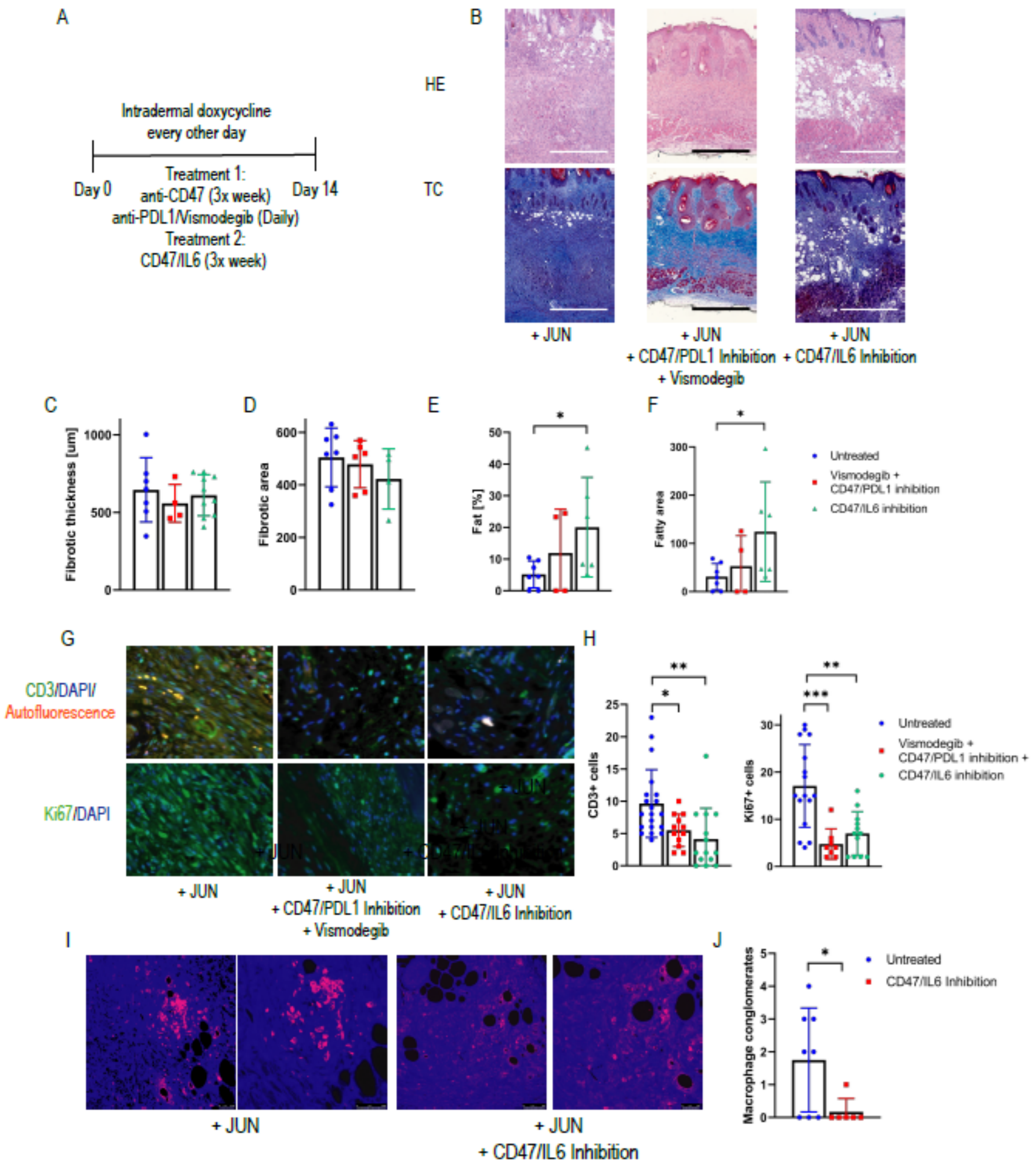

Supplementary Figure 9. Combining CD47 and IL6 inhibition prevents loss in subcutaneous fat tissue.

(A) Experimental outline

(B) Representative H&E and Trichrome skin stains of untreated mice, mice under CD47/PDL1 inhibition and vismodegib, and mice under CD47/IL6 inhibition. Scale = 500 µm. Bar graphs represent means with standard deviations.

(C) Thickness of the dermal fibrotic/connective tissue in µm. Fisher's multiple comparisons test. n=4-7. Bar graphs represent means with standard deviations.

(D) Area of the fibrotic tissue in untreated and treated samples, values indicate $\mu m^2/\mu m$ skin width. Fisher's multiple comparisons test. n=4-7. Bar graphs represent means with standard deviations.

(E) Percentage of dermal fat, compared to the overall dermal area, in treated and untreated samples. Turkey's multiple comparisons test. * $p < 0.05$. n=4-7. Bar graphs represent means with standard deviations.

(F) Area of dermal fat tissue in untreated and treated samples, values indicate $\mu m^2/\mu m$ skin width. Turkey's multiple comparisons test. * $p < 0.05$. n=4-7. Bar graphs represent means with standard deviations.

(G) Representative stains against Ki67 and CD3. Counterstains with DAPI.

(H) Quantification of CD3+ and Ki67+ stains. Indicated are the number of positive cells/high power view (63x). Turkey's multiple comparisons test. * $p < 0.05$ ** $p < 0.01$ *** $p < 0.001$. n=8-20. Bar graphs represent means with standard deviations.

(I) Representative pictures of CD11b+ cells in skin fibrosis +/- CD47/IL6 inhibition.

(J) Quantification of macrophage agglomerates determined by more than 20 macrophages/High power view in each sections. Two-sided t-test * $p < 0.05$. n=8. Bar graphs represent means with standard deviations.. .

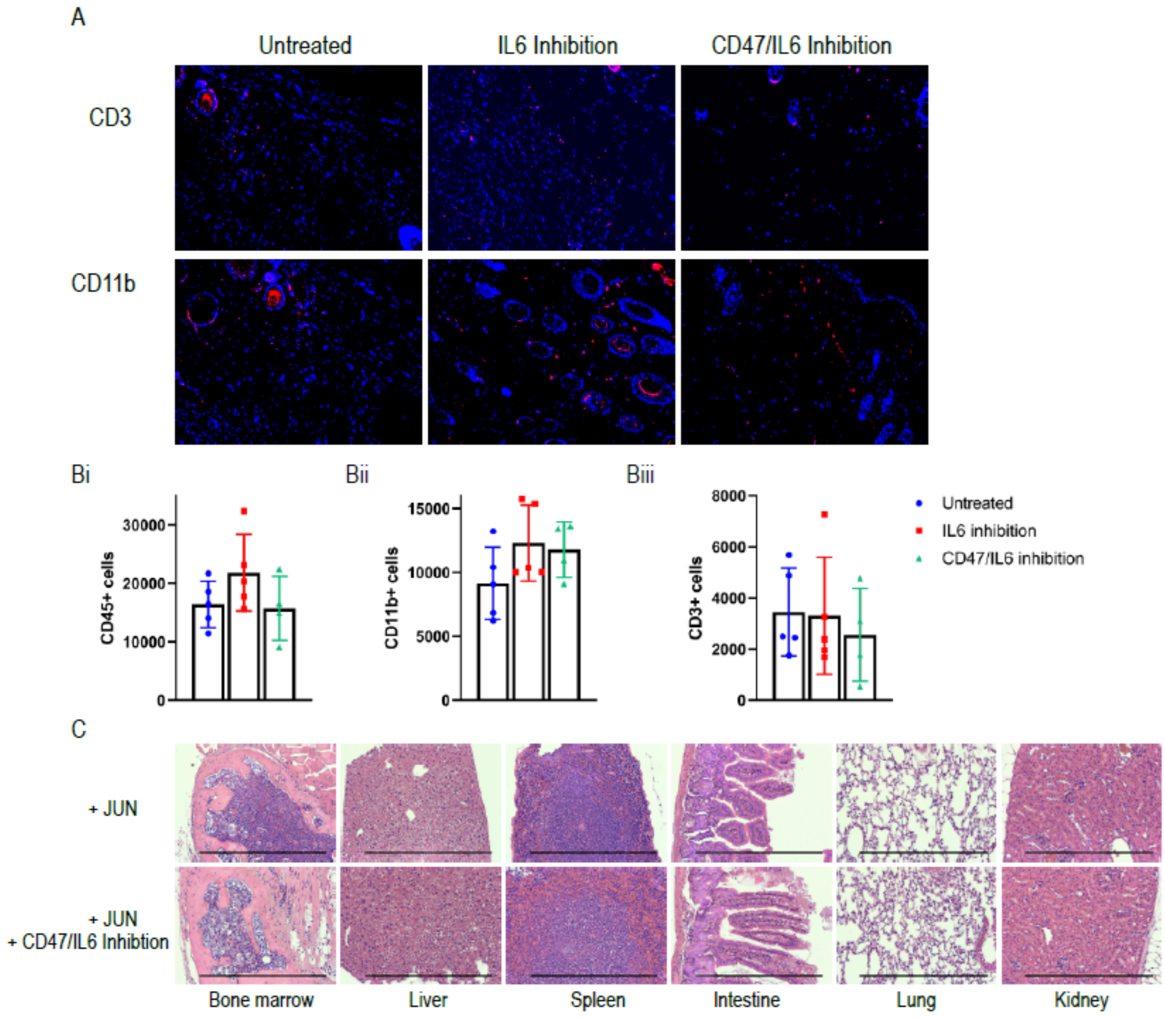

Supplementary Figure 10. Immune infiltrate in the therapeutic study.

(A) Representative immunofluorescence stains against CD3 and CD11b in the three groups (Untreated, IL6 inhibition only and CD47/IL6 inhibition)

(B) Quantification through flow cytometry for CD45+ cells. Turkey's multiple comparisons test. n=4. Bar graphs represent means with standard deviations.

(C) Quantification through flow cytometry for myeloid CD11b+ cells. Turkey's multiple comparisons test. n=4. Bar graphs represent means with standard deviations.

(D) Quantification for CD3+ T cells. Turkey's multiple comparisons test. n=4. Bar graphs represent means with standard deviations.

(E) Corresponding organ sections from the untreated and the CD47/IL6 inhibition group. Flow cytometry numbers represent number of cells/100,000 live cells.

FACS Antibodies

Antigen	Manufacturer	Catalog #	Clone	Conjugation
CD3	Biolegend	100209	17A2	APC
CD4	Biolegend	100422	GK1.5	PE-Cy7
CD11b	BD	553311	M1/70	PE
CD11c	Biolegend	117324	N418	APC-Cy7
CD25	Biolegend	102035	PC61	BV605
CD26	Biolegend	137805	H194-112	FITC
CD31	BD	553373	MEC 13.3	PE
CD45	Biolegend	103110	30-F11	Pe-Cy5
CD47	Biolegend	127527	Miap301	BV421
CD326	Biolegend	118218	G8.8	APC-Cy7
F4/80	Biolegend	123116	BM8	APC
PDL1	Biolegend	124312	10F.9G2	APC
Phospho c-Jun (Ser73)	CST	32705	D47G9	Rabbit
Sca1	Biolegend	108114	D7	Pe-Cy7
PI	Biolegend	421301		

Immunofluorescence/Immunohistochemistry antibodies

Antigen	Manufacturer	Catalog #	Clone	Dilution
Phospho c-Jun (Ser73)	CST	32705	D47G9	1:100
Collagen 1	Abcam	ab34710		1:100
CD47	R&D	AF1866		1:40
CD47	ThermoFisher	14-0479-82	B6H12	1:50
PD1	Cell marque	315M-96	NAT105	1:100
PD1	R&D	AF1021		1:40
CD68	Agilent	GA60961-2	KP1	1:200
CD31	Dako	m0823		1:150
Adiponectin	Abcam	ab22554		1:100
CD26	Abcam	ab28340		1:100
CD26	R&D	AF954		1:40
PDL1	R&D	AF1019		1:40
FSP1	MilliporeSigma	07-2274		1:200

Western Blot antibodies

Antigen	Manufacturer	Catalog #	Clone	Dilution
Phospho c-Jun (Ser73)	CST	32705	D47G9	1:1000
c-Jun	CST	91655	10F.9G2	1:1000
Phospho-Stat3 (Tyr105)	CST	9131S		1:1000
GAPDH	GeneTex	GTX627408	GT239	1:50,000
FSP1	MilliporeSigma	07-2274		1:1000

Primer sequences for qPCR

Gene	Species		Primer sequence
Gli1	Mouse	mGli1 Forward	TGGGATGAAGAAGCAGTTGGG
		mGli1 Reverse	TTGAACATGGCGTCTCAGGG
Gli2	Mouse	mGli2 Forward	GCCTCTGAGATGGAGACTTCTG
		mGli2 Reverse	TCATGTCAATCGGCAAAGGC
Gli3	Mouse	mGli3 Forward	GGAGGGTGTTCTCCTCTGAC
		mGli3 Reverse	AGGCCATCACATCCCAACTC
Actb	Mouse	mActb Forward	CTCTGGCTCCTAGCACCATGAAGA
		mActb Reverse	GTAAAACGCAGCTCAGTAACAGTCCG
Smo	Mouse	mSmo Forward	GGTTTTAATGGTGGGAGAGGGA
		mSmo Reverse	GATCGAAGCTGTCTTCAACCC
Ptch1	Mouse	mPtch1 Forward	ACAAAGCCGACTACATGCCA
		mPtch1 Reverse	TCGTAGGCCGTTGAGGTAGA
Kif7	Mouse	mKif7 Forward	ATGCCACCGTCTTTGCCTAT
		mKif7 Reverse	GTAGGATACATGCACCAGGCA

Primer for Genotyping

Gene	Primer#	Sequence
**Rosa26**	Rosa A	AAAGTCGCTCTGAGTTGTTAT
	Rosa B	GCGAAGAGTTTGTCCTCAACC
	Rosac	GGAGCGGGAGAAATGGATATG

**Jun**	ColA	GCACAGCATTGCGGACATGC
	ColB	CCCTCCATGTGTGACCAAGG
	ColC	GCAGAAGCGCGGCCGTCTGG

Supplementary Table 1. Primary antibodies and Primers.

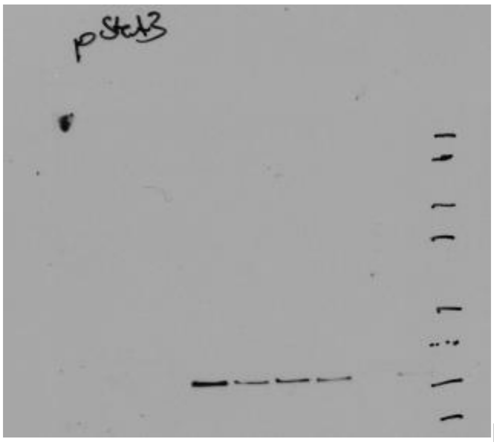

pStat3

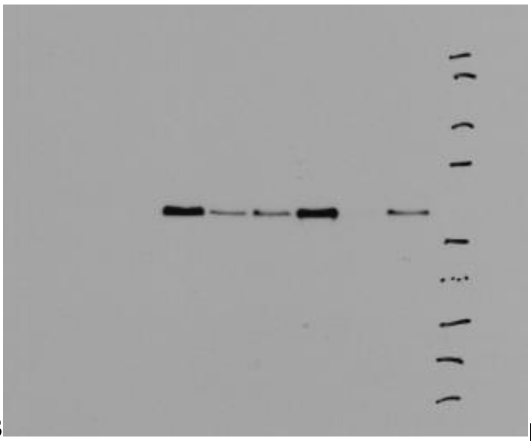
pJun

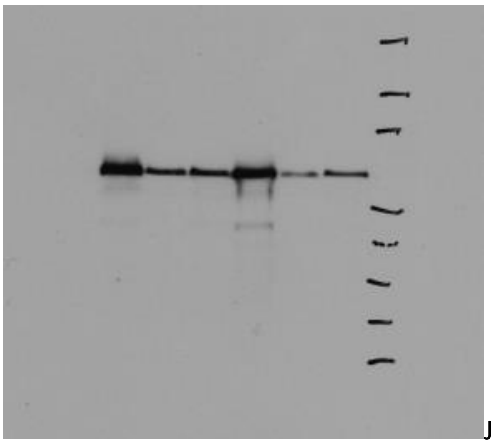
Jun

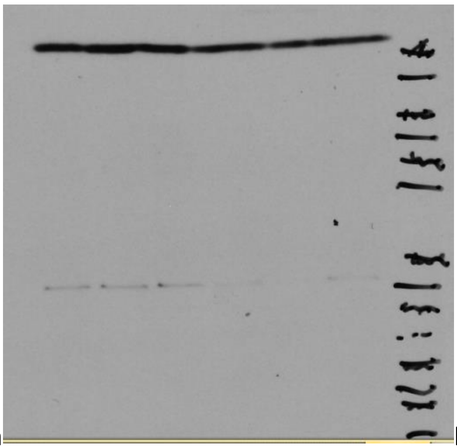
FSP

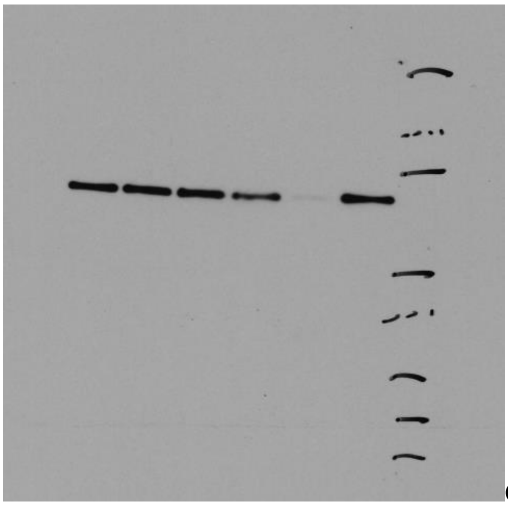
GAPDH
